# Modern Breeding Strategies and Tools for Durable Late Blight Resistance in Potato

**DOI:** 10.3390/plants13121711

**Published:** 2024-06-20

**Authors:** Ioana Virginia Berindean, Abdelmoumen Taoutaou, Soumeya Rida, Andreea Daniela Ona, Maria Floriana Stefan, Alexandru Costin, Ionut Racz, Leon Muntean

**Affiliations:** 1 Department of Crops Sciences: Genetics, Faculty of Agriculture, University of Agricultural Sciences and Veterinary Medicine Cluj-Napoca, Calea Manastur 3-5, 400372 Cluj-Napoca, Romania; ioana.berindean@usamvcluj.ro (I.V.B.);; 2 Laboratoire de Phytopathologie et Biologie Moléculaire, Département de Botanique, École Nationale, Supérieure Agronomique, Avenue Pasteur (ENSA-ES 1603), Hassan Badi, El-Harrach, Algiers 16200, Algeria; 3 Département d’Agronomie, Faculté des Sciences de la Nature et de la Vie (SNV), Université Chadli Bendjedid, BP N°73, El Tarf 36000, Algeria; 4 Department of Crops Sciences: Plant Breeding, Faculty of Agriculture, University of Agricultural Sciences and Veterinary Medicine Cluj-Napoca, Calea Manastur 3-5, 400372 Cluj-Napoca, Romania; andreea.ona@usamvcluj.ro (A.D.O.);; 5 National Institute of Research and Development for Potato and Sugar Beet Braşov, Fundaturii Street 2, 500470 Braşov, Romania

**Keywords:** potato, late blight, resistance breeding, gene pyramiding, gene editing, CRIPR-Cas9, MAS, GWAS, gene silencing, genetic transformation

## Abstract

Cultivated potato (*Solanum tuberosum*) is a major crop worldwide. It occupies the second place after cereals (corn, rice, and wheat). This important crop is threatened by the Oomycete *Phytophthora infestans*, the agent of late blight disease. This pathogen was first encountered during the Irish famine during the 1840s and is a reemerging threat to potatoes. It is mainly controlled chemically by using fungicides, but due to health and environmental concerns, the best alternative is resistance. When there is no disease, no treatment is required. In this study, we present a summary of the ongoing efforts concerning resistance breeding of potato against this devastating pathogen, *P. infestans*. This work begins with the search for and selection of resistance genes, whether they are from within or from outside the species. The genetic methods developed to date for gene mining, such as effectoromics and GWAS, provide researchers with the ability to identify genes of interest more efficiently. Once identified, these genes are cloned using molecular markers (MAS or QRL) and can then be introduced into different cultivars using somatic hybridization or recombinant DNA technology. More innovative technologies have been developed lately, such as gene editing using the CRISPR system or gene silencing, by exploiting iRNA strategies that have emerged as promising tools for managing *Phytophthora infestans*, which can be employed. Also, gene pyramiding or gene stacking, which involves the accumulation of two or more *R* genes on the same individual plant, is an innovative method that has yielded many promising results. All these advances related to the development of molecular techniques for obtaining new potato cultivars resistant to *P. infestans* can contribute not only to reducing losses in agriculture but especially to ensuring food security and safety.

## 1. Introduction

Potato is one of the major crops worldwide, being the fourth largest agricultural production following cereals (corn, rice, and wheat). In particular regions and countries, such as Algeria and Romania, it is cultivated as the primary crop. Several diseases threaten this crop and, therefore, the food safety of a large proportion of the world population, for instance, early blight and brown spot (*Alternaria solani*, *A. protenta*, *A. grandis*, *A. alternata*, and many other *Alternaria* species [1], Fusarium wilt and Fusarium dry rot (*Fusarium* sp.), *Neocosmospora solani*, [2], black scurf and rhizoctonia canker (*Rhizoctonia solani*), pink rot (*Phytophthora erytroseptica*), verticilium wilt (*Verticilium dahliae*), PVY (*Potato virus Y*), bacteria wilt (*Ralstonia solanacearum*), ring rot (*Clavibacter michiganensis* ssp. *sepedonicus*), blackleg (*Erwinia carotovora*), *Streptomyces scabies*, and late blight (*Phytophthora infestans*) [3,4,5], to cite only the most important.

**Figure 1:**
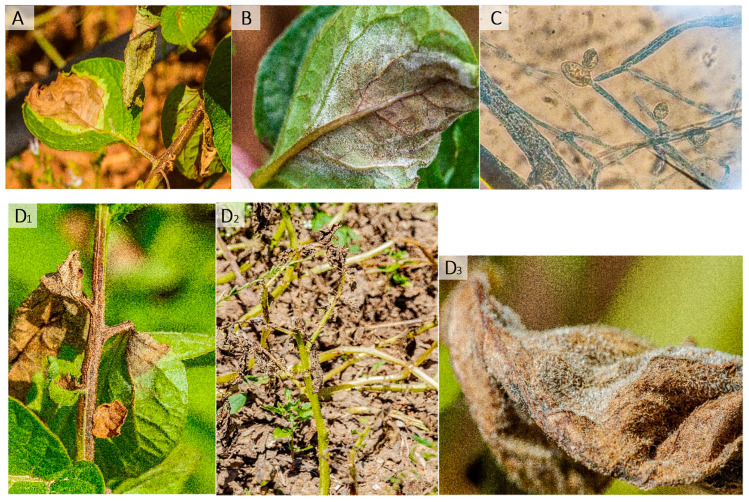
Symptoms of late blight disease. The typical symptoms of late blight: on the upper side of the leaf, an oily necrotic spot, surrounded by pale green (**A**); on the underside of the leaf: a white down is observed (**B**). This white down is the pathogens sporangiophres and sporanges (**C**). Stem and petioles could also be attacked (**D_1_**). Advanced disease is manifested by a blight of the leaves and possibly the whole plant (**D_2_**,**D_3_**). Photograph: A. Taoutaou.

By far, late blight is the most devastating potato disease worldwide [6] and is part of the downy mildews group of plant diseases. They are caused by Oomycetes and not by fungi, being fungi-like microorganisms. The disease is characterized by a necrotic lesion in the upper face of the leaf that starts as a chlorotic spot; typically, the lesion is water-soaked or oily, surrounded by a light-green-coloured edge (Figure 1A). On the underside of the leaf, white down fuzzy mycelia are observed (Figure 1B), where the sporangia are produced (Figure 1B,C). Symptoms on the stem and petioles are brown to dark necrotic lesions [6] (Figure 1D_1_–D_3_).

*P. infestans*, the Oomycete causing late blight disease, is known as the most aggressive pathogen in potato crops [7]. It originated from Mexico and now, its presence is ubiquitous, wherever potato is cultivated. The first immigration was estimated to be just before the Irish famine. The race US-1 (A1 mating-type genotype) was the only genotype that existed outside Mexico [8,9]. Then, following the immigration of the mating type A2, a huge increase in the diversity of this pathogen was observed worldwide [10,11]. Now, the beast is not confined to Mexico.

Controlling late blight disease is a never-ending war. Since its first dramatic entrance in human history with the Irish famine in the 1840s, scientists and breeders have worked to control it. Even now, after approximatively 180 years, it is a re-emerging threat to food security [12]. The actual main control tool is the excessive use of fungicides. Resistance is the best alternative to combat this disease, especially when taking into consideration concerns regarding the effects of these fungicides on humans (users and consumers both), the environment (water and soil pollution), and on the pathogen itself (development of new genotypes resistant to fungicides). Despite the huge efforts made in breeding potato with late blight resistance, it is a never-ending endeavour due to its virulence and genome structure, which highlights its quick adaptation ability and evolutionary potential [13].

The present study highlights the importance of obtaining new potato varieties resistant to *P. infestans*, from the discovery of genetic resources that can provide resistance genes to the use of modern genetic and molecular methods to introduce these genes into the crop plants’ genome or editing the genome to combat this pathogen. 

## 2. Genetic Resources for *Phytophthora infestans* Resistance

### 2.1. Potato Genetic Resources for Late Blight Resistance

Genetic resources are an essential part of any breeding program. For potato, they are concentrated on the American continent. The family of *Solanaceae* includes 90 genera and 3000–4000 species [13]. The genera *Solanum* is the largest, where the cultivated potato *S. tuberosum* is classified, as well as other important crops, such as tomato (*S. lycopersicon*) and eggplant (*S. melongena*) [13]. According to Spooner and Salas, in Gopal and Khurana [14], genera of *Solanum* are found from the southeast of the United States to Chile and Argentina. 

According to Bradshaw et al. [15], only a very small sample of the potential biodiversity has been exploited. In fact, from about 107 wild potato species [16], 32 have been used to identify and map *Rpi* genes [17]. And only a smaller number of these genes have been used in commercial cultivars. 

The classical relative that has been widely used is *S. demissum*. Potato breeding for resistance to late blight was first based on the introgression of *R* genes from this wild relative. A total of 11 *R* genes were identified (*R1*, *R2*, *R3*…, *R11*) [18,19]. The majority of these genes were overcome by the pathogen. The exception was *R8*, which, in reality, is deployed with other genes (pyramids) of *Rpi* genes [20]. Other wild relatives with a significant number of identified *Rpi* genes include the following: *S. berthaultii* (*Rpi-ber1*, *Rpi-ber2* [21], *Rpi-ber1.2*, *Rpi-ber1.3*, *Rpi-ber1.4* [22]), *S. bulbocastanum* (*Rpi-blb1*, *Rpi-blb2* [23], *Rpi-blb3* [24], *Rpi-blb4* [25]), *S. stoloniferum* (*Rpi-sto1* [25,26], *Rpi-pta1*, *Rpi-sto2*, *Rpi-pta2* [27]), *S. venturi* (*Rpi-vnt1.1* [28]). A comprehensive review about *Rpi* genes in potato to late blight is given by Paluchowska et al. [17].

The search for new *Rpi* gene sources is still ongoing. Recently, Karki et al. [29] found several new Solanum species that could be bearers of new *R* genes: *Solanum albornozii*, *S. agrimoniifolium*, *S. chomatophilum*, *S. ehrenbergii*, *S. hypacrarthrum*, *S. iopetalum*, *S. palustre*, *S. piurae*, *S. morelliforme*, *S. neocardenasii*, *S. trifidum*, and *S. stipuloideum*. Duan et al. [30] screened 189 genotypes belonging to 20 Solanum species, and they found that the genotypes highly resistant to late blight belong to five wild species, *S. bulbocastanum*, *S. cardiophyllum*, *S. jamesii*, *S. brachycarpum*, and *S. trifidum*. Rogozina et al. [31] screened the Vavilov Institute of Plant Genetic Resources collection and found out that the species with the most resistance are *S. bulbocastanum*, *S. demissum*, *S. cardiophyllum*, and *S. berthaultii*. Also, there are some potato cultivars that are resistant to late blight disease, for instance, Sarpo Mira, Bzura, Fortuna, and Azaria, which could be considered genetic resources by breeders and could be used for other breeding programs.

Conventional breeding methods present numerous obstacles in enhancing the economic traits of crops. This is due to the extensive requirement for backcrossing and rigorous selection pressure, in most cases, to incorporate beneficial traits into the germplasm, resulting in a time-consuming and labour-intensive process [32]. 

In Romania, the National Institute of Research and Development for Potato and Sugar Beet (NIRDPSB) Brasov developed ten potato varieties (Brasovia, Sarmis, Marvis, Castrum, Cosiana, Sevastia, Darilena, Ervant, Asinaria, and Foresta) with moderate resistance to leaf spot and tuber diseases. The Azaria variety is resistant to late blight (*Phytophthora infestans*) on both foliage and tubers [33]. Also, Cezarina has a high level of resistance on foliage and tubers that may be exploited and can reduce the number of treatments [34]. 

The priority of breeding programs is to enhance productivity, nutritional quality, and disease resistance. Although some of these traits can be found in wild potato relatives, incorporating them into elite cultivars can be a process that takes many years, and some of them may have a temporary effect [35].

**Figure 2:**
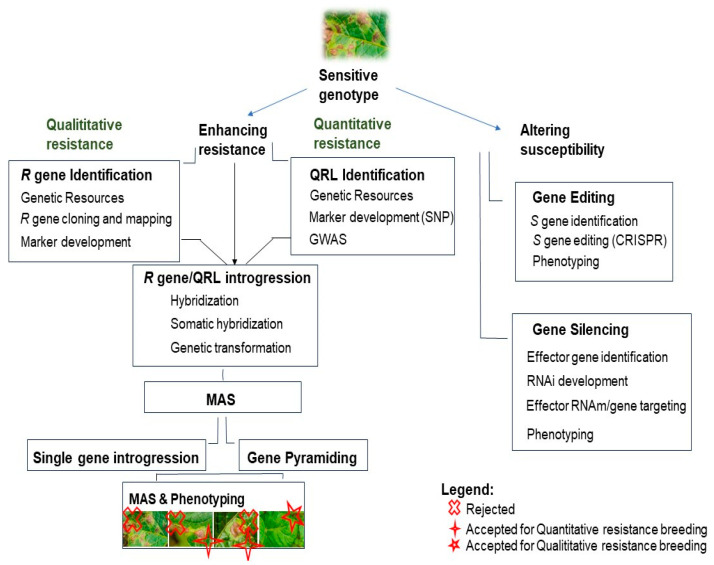
Breeding strategies of resistance for *P. infestans*. There are mainly two strategies for breeding: enhance the plant resistance and/or alter its susceptibility to the pathogen. There are two types of resistance: the first one is called qualitative and is total (the plant is or resistant, or susceptible); the second, called quantitative, is partial (the plant could have some degree of resistance/susceptibility). Legend: x: rejected for both qualitative and quantitative resistance breeding (the plant is too susceptible to the disease); +: accepted for quantitative resistance (there is an enhancement of the resistance: the plant is more resistant than the starting material); ✩: Accepted for qualitative resistance: the plant is totally resistant to the pathogen, and there is no sign of the disease.

Breeding for resistance begins with identifying genetic resources that possess the desired resistance traits. This is followed by searching for resistance genes through methods like gene mining and GWAS, and then identifying and cloning these genes using techniques such as MAS and QRL. The next step involves gene introgression into potato cultivars via natural and somatic hybridization or recombinant DNA technologies. While some breeders use a single R gene, R gene stacking is now recommended. Additionally, susceptibility genes can be edited using gene editing technologies like CRISPR-Cas9 (Figure 2).

### 2.2. Resistance Genes to Late Blight

*P. infestans* is a hemibiotrophic pathogen, which means that it has two stages of infection. For the first step of infection, it behaves like a biotrophic pathogen; then, it acts as a necrotrophic one in the second infectious stage. The symptoms described above are seen only in the second stage. 

The resistance to this type of pathogen is thought to follow the gene-for-gene model developed by Flor (1971) [36]. This model says that for each gene of avirulence of the pathogen, there is a gene of resistance in the plant. Now, we know that effectors (avirulence factors) are detected and recognized by resistance proteins. They function as a surveillance system and are responsible for *P. infestans* detection and recognition. The role of R protein is to detect and recognize the pathogen, by recognizing its effectors. The *P. infestans* effectors that are recognized by R proteins are called RXLR effectors. Following effector detection, a signal is transduced to the nucleus, inducing the expression of genes associated with resistance, such as genes of pathogenesis-related protein (PR proteins), genes involved in the synthesis of secondary metabolites that are toxic to *P. infestans*, and, finally, those involved in the hypersensitive reaction (HR). This is called effector-triggered immunity (ETI). 

Another type of resistance is called PAMP (pathogen-associated molecular pattern)-triggered immunity (PTI). It is induced as a result of the detection of elicitors. Elicitors can originate from the pathogen, such as chitin fragments in fungi or *INF1* elicin produced by *P. infestans*. Another type of elicitor is damage-associated molecular patterns (DAMPs). Those are molecules originating from the plant under the action of the pathogen, such as cutin monomers resulting from the digestion of the plant cell wall by pathogen cell wall-degrading enzymes (here, cutinases). This kind of DAMP is called constitutive DAMPs. The second type of DAMPs is induced DAMPs; these elicitors are produced by the plant following pathogen detection, for instance, Systemin, Hypsys, Nep1, etc. Elicitors are detected by specialized receptors on the plant membrane cell, called Pattern Recognition Receptors (PRR) [37,38].

Plant resistance genes are classified mainly into five classes, according to Bezerra-Neto et al. [39], based on their conserved domains. The first is the NLR (Nucleotide binding-site Leucine rich-repeat) class. It is the largest class, and the majority of plant *R* genes belong to it. It is divided into two subfamilies: CC-NLR and TIR-NLR. The second is TM-LRR, the third is RLK (Receptor-Like Kinase), the fourth is STK (Serine/Threonine Kinase), and the fifth is CC (Coiled Coil). Classes II, III, and V are located in the plant cytoplasmic membrane, and classes I and IV are cytoplasmic.

**Figure 3:**
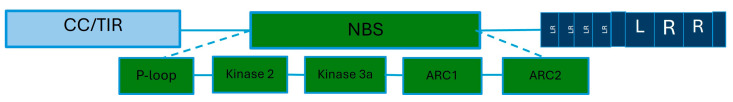
Schematic representation of the structure of a resistance gene of potato to late blight disease. *R* genes are composed of three major conserved domains: the first one can be a coiled or Toll Interleukin domain. In the middle is a Nucleotide binding site. This domain also contains several conserved regions: P-loop, Kinase2, Kinase 3a, ARC1, and ARC2. On the other end, the Leucine rich-repeat domain is found. Legend: CC: coiled coil, TIR: Tall Interleukin 1 Receptors, NBS: Nucleotide binding site, LR: Leucine rich, LRR: Leucine rich-repeat.

There are more than 70 potato genes of resistance to *P. infestans* identified [17]. These genes contain highly conserved domains of Leucine rich-repeat (LRR) and Nucleotide binding site (NBS). Some also have a coiled coil [40], and they are part of the first class (NLR) (Figure 3). 

Based on the number of isolates a gene can offer resistance against, two types of *R* genes have been discovered:

- *Broad (large)-spectrum R* genes: they offer resistance against a wide range of *P. infestans* genotypes. The flagship of this category is *Rpi-blb1* (known also as *RB*), identified at first in the wild species *S. bulbocastanum* [23,41]. They are able to recognize genus-specific or species-specific effectors. Other genes were discovered later: *Rpi-blb2* and *Rpi-blb3* [23,42]. Homologues of *RB* were also discovered in other relative wild species: *S. stoloniferum* (*Rpi-sto1*) [27]. These genes are supposed to offer resistance against all *P. infestans* species. However, some have reported that the isolate NL13316 can break this resistance, so it is qualified it as an *RB* virulent isolate [29].

On the other hand, some of these genes offer resistance to different species (not only races) by recognizing a conserved effector in the genera or family. Lin et al. [43] identified an *R* gene (*Rpi-amr3*) from *S. americanum* (wild species) that offers resistance against multiple *Phytophthora* species: *P. parasitica* and *P. palmivora*. *Rpi-amr3* recognizes the effector AVRamr3. An RXLR-WY effector is conserved in *Phytophthora* species.

- *Narrow-spectrum R* genes: these are genes that offer resistance to only one race; in fact, they recognize race-specific effectors. This group is represented by the majority of *Rpi*, such as *R1*, *R2*, and *R3* from *S. demissum*, and other species.

This resistance, based on a single or a small number of dominant *R* genes, is called qualitative resistance, and it offers immunity or total resistance against this pathogen. However, it is easily breakable. The other type of resistance is called quantitative resistance, which is not total, and it is controlled by several genes, each one contributing somewhat to resistance. This resistance is considered durable. For breeding this type of resistance, breeders look for quantitative resistance loci (QRLs) (Figure 2).

Susceptibility is influenced not only by the absence of *R* genes but also by the activity of susceptibility (S) genes. *S* genes facilitate infection and support compatibility between the pathogen and the host plant, giving pathogens an advantage in colonizing host plants [44]. According to the referenced authors, *S* genes can potentially be utilized in breeding programs aimed at developing resistant plant varieties. They classified *S* genes into three major groups based on their roles during different stages of infection: (1) early pathogen establishment: genes active during the initial phase of pathogen infection; (2) modulation of host defences: genes involved in altering the plant’s defence mechanisms; (3) pathogen sustenance: genes that help sustain the pathogen within the host [44]. The primary focus is on the dual roles of *R* and *S* genes in plant resistance and susceptibility, emphasizing the potential for manipulating *S* genes to breed resistant plants.

### 2.3. Effectoromics—A New Method of R Gene Mining

Effectors are pathogen tools that infect a host plant. They are molecules produced by the pathogen to manipulate host physiology in the scope of promoting infection; when detected by the plant R proteins, they trigger defence responses [45]. Plant R proteins have the duty to identify and recognize the pathogen by recognizing its effectors. Plant R proteins can interact directly with effectors (direct recognition) or indirectly, by recognizing the effect of the effector on its target (indirectly). In some cases, R proteins recognize the alterations in decoy molecules, produced by the plant, of the effector’s target. The recognition of the effector is microscopically manifested through the hypersensitive reaction. As a consequence, effectors can be used in the process of screening the potato germplasm looking for *Rpi* genes. This approach is called effectoromics. 

Effectoromics, as defined by Du and Vleeshouwers [46], is a “*functional genomics approach that uses effectors to probe plant germplasm to detect R genes*”. Since the sequencing of the *P. infestans* genome in 2009, and with the use of bioinformatics, this approach has become possible, with great potential to accelerate *R* gene identification and cloning and the identification of *R* gene homologs, thus aiding to overcome the natural barriers of hybridization by looking for these homologs in compatible species, as well as detecting specificity and even helping with *R* gene deployment [47]. Effectors could be used as functional markers and are accelerating and improving resistance breeding [48].

Effectoromics was developed first for the identification of potato *Rpi* genes [49]. The reference genome of *P. infestans* harbours more than 560 predicted *RXLR* and 190 *CRN* effector genes. Vleeshouwers et al. [26] used effectoromics to identify *Rpi-sto1* and *Rpi-pta1* homologs of *Rpi-blb1*. Weymers et al. [50] used this approach to identify the *Rpi-vnt1.1* in *S. okadae*. The resistance genes of the resistant cultivar Sarpo Mira, *Rpi-Smira1* and *Rpi-Smira2*, were identified using this approach [51]. More recently, Lin et al. [43] estimated that the reference genome of *S. americanum* contains approximately 550 *R* genes. Then, they successfully identified and cloned the *Rpi* gene *Rpi-Amr4* (from *S. americanum*) using the effectoromics approach. It is worth noting that this approach could be transferred to other pathosystems to enhance the gene mining efficiency. For instance, Giesbers et al. [52] used this approach to identify an *R* gene of lettuce against *Blumeria lactucae*. 

## 3. Genetic Methodologies for Breeding Resistance to *Phytophthora infestans*

### 3.1. Marker-Assisted Selection for Phytophthora-Resistant Potato Plants

Marker-assisted selection (MAS) is a revolutionary technique that has transformed the field of plant breeding by allowing for the rapid and precise selection of desirable traits in crops [53]. MAS provides a more efficient and precise method for selecting disease resistance [54] and offers a more efficient and targeted approach, utilizing molecular markers linked to resistance genes to expedite the breeding process [55]. MAS allows for the selection of plant traits based on the presence of specific DNA markers associated with those traits [54]. 

In recent years, molecular markers have been used in the agronomic sciences as powerful tools for analysing genetic variation, as they efficiently link phenotypic and genotypic variation [56]. In potato, molecular markers are used in studies on cultivar identification [57], genotype characterization and genetic diversity [58,59], resistance to biotic and abiotic stress [60], pathogen resistance [61,62], and trait mapping [63]. This technique has significantly accelerated the breeding process [56], because it allows for the identification of resistant plants at an early stage, enabling faster and more targeted breeding programs [64]. Overall, the application of MAS in potato breeding holds great potential in creating resilient and high-yielding cultivars that can combat the destructive effects of late blight disease on potato crops [65]. MAS has been successfully used in improvement programs for the development of potato varieties resistant to late blight caused by *P. infestans* [66].

Late blight resistance has been the focus of continuous and intensive research for over a century, as *P. infestans* has the ability to rapidly mutate to evade plant defences, resulting in the most destructive disease, known as potato late blight [7]. While single *R* genes can be easily overcome by the rapidly evolving pathogen, it is now necessary to combine multiple *R* genes and/or quantitative trait loci (QTLs) against *P. infestans* to breed potato cultivars with durable resistance. This approach could prolong the rate of late blight resistance [61]. Conventional breeding methods are crucial but time-consuming (10–15 years) due to several generations of backcrossing, field trials, and phenotype selection. This process is challenging in potatoes because of their polyploidy (2n = 4x = 48), tetrasomic inheritance, and chromatid segregation [61]. Alternatively, the use of molecular markers for genotype analysis to identify and select species with desired traits is becoming more prevalent [67]. This selection approach, known as marker-assisted selection (MAS), offers a considerably more cost-effective selection method per cultivar in potato breeding compared to traditional field-based phenotypic screening [68]. Molecular markers and genome sequencing data are expected to be pivotal in MAS. Key factors to consider for markers in MAS are their ease of use, reliability, cost-effectiveness, linkage to genes controlling the trait of interest, and the amount of phenotypic variation explained by the marker [69]. 

Most of the molecular markers used previously in mapping were either restriction fragment-length polymorphism (RFLP) or amplified fragment-length polymorphism (AFLP), found exclusively using diploid-segregating populations. RFLP was the initial molecular marker method and the only marker system founded on hybridization. Within a species, individuals show polymorphism due to insertions/deletions, point mutations, translocations, duplications, and inversions [70]. AFLP markers are generated using a technique that involves digesting genomic DNA with restriction enzymes, followed by selective PCR amplification of the resulting fragments [71]. AFLP markers are reliable but expensive, labour-intensive, and have a long assay technique, whereas RFLP and simple sequence repeat (SSR) systems are based on the polyacrylamide gel system, a long and laborious technique. Therefore, these markers are not easily assayed and are, consequently, unsuitable for MAS [61]. Conversely, Sequence-Characterized Amplified Region (SCAR) and Cleaved Amplified Polymorphic Sequence (CAPS) markers are straightforward to use. SCAR markers exploit polymorphisms in the primer sites, leading to the absence or presence of an amplified band [72], while CAPS markers utilize a restriction site polymorphism following PCR amplification [61,73].

In studies on late blight resistance, different types of markers were developed and used for targeting resistance genes. According to the bibliography and based on different research on *R* genes in wild species of potato, the SCAR marker CosA can be used to detect the resistance gene *R1* [69]. The *R2* gene can be targeted through the SSR marker R2 [74]; the *R3b* gene can be identified through the marker R3b [75] and the SSR marker R3b4 [76]. *Rpi-blb1* can be targeted either by SCAR markers like Rb-1223 [61] and RB-629/638 [75] or SSR markers such as BLB1 [77] and 1521/518 [78]. In Jacobs et al. [79], the CAPS marker CP58 (MspI) was linked to the resistance gene *Rpi-cap* and OPA17. A RAPD marker was used to detect the QTL_phu-stn [80].

Thanks to advances in high-throughput genotyping technologies, such as genotyping by sequencing (GBS), dense markers have been discovered for detecting disease resistance and other important traits in potatoes. This approach has led to the identification and filtration of a substantial number of SNP markers, ultimately resulting in the creation of a high-quality subset [67]. Syverson and Bradeen [81] mentioned that single nucleotide polymorphisms (SNPs) represent the ultimate molecular marker type that continued to evolve against *P. infestans*. DNA sequence-based markers like SNPs are the most common form of genomic DNA variation and are preferred over other genetic markers due to their low mutation rate, ease of use, and cost-effectiveness for genotyping [82]. Currently, genome-wide association studies (GWASs) are conducted using SNP markers to locate genes/QTLs linked to resistance against late blight in potato [83,84]. The continuous development in the molecular markers’ technology from RFLP to SNPs and a diversity of array-technology-based markers have revolutionized the study and breeding of late blight resistance in potatoes. Marker-assisted selection has enabled breeders to introgress multiple resistance genes and quantitative trait loci, enhancing the durability and effectiveness of late blight resistance in potato cultivars.

Despite these challenges, the potential benefits of using MAS in potato plant breeding, particularly in the context of disease resistance, make it a valuable tool for improving crop productivity and sustainability [85].

### 3.2. Genome-Wide Association Study

Genome-wide association studies (GWASs) are a widely adopted technique to unravel genotype–phenotype associations in many species due to advances in Next-Generation Sequencing (NGS) technologies [86]. GWAS methods assess the statistical correlation between markers, typically single nucleotide polymorphisms (SNPs), and the phenotype of a target trait observed in large panels of individuals (natural populations) or lines (diverse germplasm collections), which are often genetically distant [87]. Genome-wide association studies are carried out by analysing the entire genome for significant associations between a panel of SNPs and a specific phenotype. These associations are subsequently confirmed independently to demonstrate that they (a) contribute directly to the parameter of interest or (b) are linked to a particular phenotype [88]. Phenotypic variation, population size and structure, allele frequency, and linkage disequilibrium collectively influence the power of GWASs [87].

The GWAS has emerged as a powerful approach for dissecting the genetic basis of complex traits by identifying specific regions in the genome, known as quantitative trait loci (QTLs), which affect the phenotype. This is achieved by using sufficient markers and linkage disequilibrium (LD) between alleles [89,90]. LD refers to the non-random association of alleles at different loci [91]. The rate of LD decay determines the minimum number of markers required for a GWAS and also influences the feasibility of tagging QTLs associated with traits of interest. A low level of LD allows for more precise mapping, as it highlights only those genetic locations that are closely linked to the gene being studied and are strongly associated with the trait of interest. Achieving such precision, however, requires a larger number of molecular markers compared to situations with higher LD [92]. On the other side, ignoring the non-random association among alleles at different loci negatively affects the mapping resolution, because both causal and non-causal alleles will be included in the following analyses, potentially leading to false associations [87]. In Vos et al. [93] and Sharma et al. [94], moderate LD decay at a distance of 70 Kb to 2 Mb has been reported in the cultivated gene pool of European and North American potato.

Conducting a GWAS can be relatively easier in crops that have well-characterized genomes, extensive genetic diversity, and traits that are controlled by relatively few genes with large effects, which is why *Zea mays* L. and *Arabidopsis thaliana* L. are frequently featured in GWAS reports. A number of candidate genes and associated SNPs have been identified for various agronomic and quality traits as well as for biotic and abiotic stress tolerance, such as head smut tolerance [83], drought tolerance [95], cold tolerance [96,97], and resistance to corn borer in maize [98].

Resistance to late blight involves a complex interplay of genetic factors that contribute to the overall level of resistance observed in different potato varieties [47] because this resistance is controlled by multiple genes, and its expression is influenced by environmental factors, so its improvement is difficult. Genetic mapping is a powerful strategy that exploits genomic information to dissect such complex traits and identifies genetic determinants that may lead to crop improvement [99]. Being highly heterozygous and auto-tetraploid, the genetic inheritance in cultivated potatoes is quite complex, which complicates gene mapping. Therefore, conducting a GWAS on potatoes can be challenging. For molecular map construction and linkage analysis, diploid individuals are frequently used as parents to limit the complexity of potato genetics [100]. Several late blight resistance loci were identified using bi-parental populations at the diploid level in early genetic mapping studies [61]. However, this method does not allow for the evaluation of large gene pools or interactions involving multiple alleles that affect traits in polyploids. Recent advancements have been achieved in creating algorithms and software for genotype identification, linkage, and QTL analysis in polyploid species [101]. The identification of QTLs in autopolyploids is facilitated by innovative tools like GWASpoly, which consider the effects of allele dosage [102]. As mentioned earlier, a GWAS requires high-density molecular markers, especially single nucleotide polymorphisms (SNPs) spread across the genome. Although 8 K, 12 K, and 20 K SNPs arrays have been available in potatoes [103,104], they were developed using a specific germplasm.

Knowledge of the candidate genes and QTLs governing resistance (QRL) could accelerate the breeding process and enhance the genetic gain in potato breeding [84]. Since the availability of the genome of potato (*S. tuberosum* L., NCBI:txid4113) published in 2011 by the Potato Genome Sequencing Consortium (PGSC) using a whole-genome shotgun sequencing approach (The Potato Genome Sequencing Consortium, 2011), genetic analyses conducted on potato have been enhanced. Due to access to advanced sequencing technologies and enhanced software, the creation of a high-quality, long-read, chromosome-scale assembly and improved annotation dataset for the reference genotype of potato has been enabled [105]. Currently, the identification of candidate genes associated with various traits in potato is facilitated using the PGSC potato genome sequence portal (http://spuddb.uga.edu/dm_v6_1_download.shtml, accessed on 16 March 2024). These advancements collectively increase the value of genomic analysis in tetraploid potatoes, making it more relevant for evolutionary and breeding research.

GWASs have been successfully used to reveal genomic regions, candidate genes, and QRLs governing resistance to late blight in potatoes. In Wang et al. [83], a GWAS was conducted for late blight R traits using the AFSM sequencing approach on 284 potato cultivars from China, Australia, Belarus, Canada, Britain, the International Potato Center (CIP), Israel, Netherlands, Russia, and the United States. The main objective of the study was to dissect the genetic architecture of the cultivars and to evaluate their genetic diversity, identify loci, and molecular markers associated with the late blight R trait and to identify candidate genes for potato-breeding improvements. GWAS analysis revealed 964 loci significantly associated with the late blight R trait. The genomic regions identified harboured 14 candidate genes for late blight R traits, including genes encoding the pathogenesis-related protein, chitinase 1, R protein, protein kinase, ethylene-responsive transcription factor, and other potential plant resistance-related proteins. Lindqvist-Kreuze et al. [101] conducted a GWAS on foliar late blight resistance in tetraploid potato, using a panel consisting of 380 genotypes bred at the International Potato Center in Peru. According to the GWAS results obtained, QRL with the largest effect was identified in chromosome 9, and the highest numbers of SNPs associated to late blight resistance were mapped between 59 and 61.2 Mbp of chromosome 9 in a region that had been previously associated with late blight resistance [106].

A recent GWAS study conducted by Sood et al. [84], on 367 potato accessions, including Indian and exotic varieties and advanced breeding lines, identified several resistant elite donor lines for late blight, which could be used in introgression breeding. Eight QRLs were found for late blight, detected by additive and simplex dominance models of GWAS. The highest number of QRLs mapped on chromosome 11 indicated that it was the hotspot for late blight resistance in potatoes. This region was previously reported as the hotspot disease *R* genes in potato [107]. The major genes for late blight defence identified in the study were functionally related to response regulators, proteins of unknown function, and plant U-box and ENTH/VHS/GAT family protein.

The application of GWAS in potato has resulted in the successful identification of genomic regions associated with late blight resistance; this information can be used to develop improved varieties with enhanced resistance to late blight through marker-assisted selection or gene editing techniques.

### 3.3. DNA Methylation 

DNA methylation is a biochemical process that involves adding a methyl group to the DNA molecule, typically at the fifth carbon of the cytosine ring within CpG dinucleotides (the regions where a cytosine nucleotide is followed by a guanine nucleotide in the linear sequence of bases). This process is catalysed by enzymes known as DNA methyltransferases (Dnmts), which transfer a methyl group from S-adenosyl methionine (SAM) to the cytosine, forming 5-methylcytosine (5 mC) [108]. DNA methylation is essential for silencing retroviral elements and regulating tissue-specific gene expression. 

DNA methylation was employed for the first time in a non-model plant population, specifically cassava, revealing an average detection rate of one methylation site per 51 base pairs [109]. Drozda et al. [110] investigated DNA methylation in potato, focusing on two genotypes (‘Sarpo Mira’ and the breeding line TG 97-411), both known for high effector-triggered immunity (ETI) resistance against an avirulent *P. infestans* isolate. Their findings demonstrated that the pathogen swiftly induced 5-methylcytosine (5-mC) DNA hypermethylation in both potato genotypes. Furthermore, treatment with S-nitrosoglutathione reductase (GSNOR) led to a significant increase in the global methylation level. 

GSNOR activity is necessary to regulate the transmethylation cell activity associated with DNA (de)methylation involved in stress-responsive gene regulation [110]. Additionally, Kuznickii et al. [111] explored the role of DNA methylation and demethylation in potatoes in enhancing resistance against the virulent *P. infestans* MP977 by studying BABA-primed changes linked to transcriptional memory.

### 3.4. Genetic Engineering and Genetic Transformation of Potato

In the last two decades, the conventional potato breeding program has been increasingly improved and replaced by various methods based on genetic transformation. Hence, genetic engineering offers the chance to incorporate desired genes without modifying the allelic combinations that define prosperous commercial cultivars [35]. The goal of genetic engineering is to create organisms with desired traits or characteristics that may not occur naturally or may be difficult to achieve through traditional breeding methods. Genetic engineering can involve manipulating DNA at the molecular level to introduce, delete, or modify specific genes.

The development of this field is due to biotechnology (manipulation of single-cell cultures or organized tissues), which provides the basic substrate for the practical use of genetic engineering for potato breeding [112]. Genetic transformation is a process where numerous identical clones of the *R* gene are transferred to another bacterium called *Agrobacterium tumefaciens*. This bacterium’s natural ability is used to transfer the cloned *R* gene (or a cassette of several *R* genes) to individual cells of potato leaves, in a liquid suspension to induce transformation [113].

Genetic engineering consists of two types of gene intrograssion: trans-genesis and cis-genesis. Trans-genesis is the process of introducing a foreign gene or genes, from a sexually incompatible species, such as bacteria, into an organism’s genome, resulting in the expression of traits encoded by those genes [114]. This technique is commonly used in genetic engineering to confer new traits or characteristics to organisms for various purposes, such as improving crop yield, enhancing resistance to pests or diseases, or producing valuable proteins. Cis-genesis refers to a genetic engineering approach where genes are transferred from sexually compatible species, typically within the same genus, without introducing genes from unrelated organisms. In cis-genesis, the transferred genes are copies of genes naturally found in the target species. This technique ensures that only genetic material native to the target species is introduced, without incorporating foreign genetic elements [115].

For potatoes, many studies have revealed the importance of accelerating the breeding process or gene transfer for resistance to the late blight pathogen. In the past 15 years, research has been conducted to identify and clone multiple genes associated with resistance to the pathogen *P. infestans* in wild potato species.

Although potato is a tetraploid and a heterozygous crop, its genetics is quite challenging to understand. For this reason, due to modern technologies, genome sequencing began in 2005–2006 and continued until 2011, as part of a program called the Potato Genome Sequencing Consortium (PGSC). For this purpose, a heterozygous diploid crossing line of the *S. tuberosum* group Tuberosum (RH89-039-16) and a double-monoploid-derived genotype of the *S. tuberosum* group Phureja (DM1-3 516 R44) were used. After the process was completed, the sequence data unveiled a genome size of 844 Mb and approximately 39,031 protein-coding genes in potatoes [116].

Since the first genetically modified (GM)/biotech crop plants were introduced in the mid-1990s, from then, there has been a continuous growth in the amount of land used to plant and harvest these crops globally every year in the agriculture industry [117]. *Rpi* genes have been identified in wild potato species and transferred into cultivated potato varieties through classical breeding. A case of incorporating *Rpi* genes into the potato gene pool was demonstrated in the Bioimpuls project, which, after 11 years, led to the development of a true seed population containing single or multiple *Rpi* genes for late blight resistance through classical breeding methods [66]. 

However, the breeding process is long and difficult (up to 30–50 years) due to differences in ploidy levels between wild species and cultivated tetraploid potatoes. Significant advancements have been achieved over the last 25 years in understanding the molecular foundations of plant disease resistance mechanisms and how pathogens overcome them [118]. From “Bionica” and “Toluca” late blight resistant varieties, which bear the *R* genes, transferred with original interspecific hybridization from *S. bulbocastanum*, a process that took a long time (45 years) [119], in 2014, a new potato genotype for blight resistance was developed by inserting the gene *Rpi-vnt1.1* (from *S. venturi*) in the Desirée cultivar, by a team of British scientists. This gene is known to confer resistance to race 13_A2 and 6_A1 in detached leaf assays [118]. Also, it was demonstrated that the three *Rpi* genes (*Rpi-vnt1.1*, *RB* and *Rpi-blb2*) isolated from the wild species *S. bulbocastanum* and *S. venturii* can confer extreme, possibly stable and durable, resistance to late blight in the potato varieties ‘Tigoni’ and ‘Shangi’ cultivated in east Africa [120]. Haverkort and his team [113], as part of the DuRPh project (2006–2015), utilized cis-genesis to clone and transfer *Rpi* genes from crossable wild potato species (cisgenes) using *A. tumefaciens*, while excluding non-potato genes. 

The same genes (*Rpi--vnt1.1* and *Rpi-blb2*) was transferred into Desirée and Victoria cultivars, by electroporation, using the *A. tumefaciens* hypervirulent strain EHA105, providing complete resistance in the field over several seasons [119]. The characteristics of the local pathogen population indicate that the resistance to late blight could endure for an extended period due to its limited diversity, comprising the singular lineage 2_A1 [119].

Currently, globally, 51 transgenic events are approved, with the majority prepared by *A. tumefaciens*. Among these, 38% of the events exhibit resistance to various potato-specific diseases. These transgenic events were developed by the company J.R. Simplot Co [121]. There are four GM events with resistance to late blight, all approved for cultivation in America and Canada. These are Gen2-Z6 (Simplot Innate), X17 (Innate Acclimate), Y9 (Innate Hibernate), and W8. All GM potatoes have *Rpi-vnt1*. All these events are suitable for commercialization for food and feed in a few countries, like Australia, Japan, New Zealand, Malaysia, Philippines, and Singapore. In the European market, currently, the transgenic potato is not allowed. 

In China, major food companies have refused to use genetically modified (GM) potatoes in their processed potato products [122]. Recent milestones in Asian countries to mitigate or manage late blight in potatoes include significant advancements in genetic engineering and biotech approaches. The only country in Asia known for producing genetically modified (GM) potatoes is India. Researchers from the Central Potato Research Institute (CPRI) in Shimla developed GM potatoes (KJ66) that are resistant to late blight, aiming to improve crop resilience and reduce losses due to disease. The KJ66 potato hybrid was derived from the wild Mexican potato, and it is being developed to fight the infamous pathogen *Phytophthora infestans* (https://forumias.com/blog/transgenic-crops-in-india-need-and-challenges/, accessed on 28 May 2024). 

The GE Katahdin event SP951, expressing the *RB* gene resistant to *P. infestans*, was donated to the ICABIOGRAD (Indonesian Center for Agricultural Biotechnology and Genetic Resources Research and Development) by USAID from Cornell University, USA. This material is intended to be used as a source of resistance to late blight in the breeding program [123]. 

No GM potato has been approved as food or feed in the Republic of Korea as the country adheres to a zero-tolerance policy to unauthorized genetically modified organisms (GMOs) [124].

In conclusion, all genetic modification techniques are able to develop potatoes with elevated levels of late blight resistance in a short period, a hard thing to achieve using conventional plant breeding alone.

### 3.5. Somatic Hybridization

Somatic hybridization (SH) is the fusion of two protoplasts. The cell walls of the cells are digested, and then the resulting protoplasts are subjected to an electric pulse. It offers researchers and breeders the possibility to overcome the barriers to natural hybridization between different species and also the fears of consumers linked to the introgression of genes via the recombinant DNA technologies. 

SH represents a significant advantage in terms of time and effort for breeders, particularly in species where natural barriers impede conventional hybridization. The best case is the species *S. tuberosum* and *S. bulbocastanum*. According to Haverkort et al. [125], the process of introgressing the *Rpi-blb2* gene into *S. tuberosum* commenced in 1959 and culminated in the development of the cultivars Toluca in 2006 and Bionica in 2008, spanning a minimum of 47 years.

Somatic hybridization emerges as a particularly advantageous strategy in breeding programs aimed at achieving broad-spectrum and durable resistance. The *Rpi* genes of this type are found mainly in *S. bulbocastanum* (*Rpi-blb1*, *Rpi-blb2*, *Rpi-blb3*, *Rpi-bt1*, and *Rpi-blb4*). Somatic hybridization provides the most rapid pathway for incorporating these genes. Helgeson et al. [126] demonstrated the successful transfer of resistance to *P. infestans* from *S. bulbocastanum* into cultivated potato through SH. Rakosy-Taican et al. [127] used SH to create a potato genotype carrying a pyramid of *Rpi-blb1*, *Rpi-blb3*, *R3a*, and *R3b*. Thieme et al. [128] also used SH between wild species (*S. cardiophyllum*, *S. tarnii*, *S. etuberosum*) and cultivated potato to transfer resistance to late blight and PVY. Shi et al. [129] used SH to cross *S. tuberosum* and *S. cardiophyllum* (resistant to late blight). Also, Sedlák et al. [130] achieved late blight resistance in hybrids by employing SH techniques in crosses between *S. tuberosum* and *S. bulbocastanum*.

### 3.6. Gene Silencing

In natural plant–pathogen interactions, there is always a trans-kingdom exchange of small RNAs (sRNA), also called RNA interference (iRNA). Pathogens, including *P. infestans*, use RNAi to interfere with plant defences. 

RNA interference (RNAi) was used by Sun et al. [131] to knock down StDND1, and they found that the plants were more resistant to *P. infestans*. In 12 of the transformants, dwarfing appeared, a phenomenon that may result from either the specificity of the RNAi method used to silence the gene or variations in the efficiency of gene silencing throughout the plant’s development. Using the RNAi method to silence one or a group of genes can cause undesirable phenotypes that may produce uneven results [131]. 

Kieu and his team [132] found that knocking out the *StMLO1* gene in potatoes did not enhance late blight resistance in MLO potatoes. All eight *StMLO1* mutant lines were as susceptible to late blight disease as the wild type, Desirée. Sanju et al. [133] silenced the *Avr3a* gene of *P. infestans*, resulting in moderate gene suppression and moderate disease resistance observed in transgenic plants. This implies the need to assess the collective influence of effector genes in conjunction with the *Avr3a* gene. Orthologs of *Arabidopsis S* genes (*DND1*, *DMR6*, *DMR1*, and *PMR4*) were silenced by RNAi in potato and tomato, by Sun et al. [134]. The mutant lines of *A. thaliana*, Dnd1 plants, also demonstrate improved resistance against a wide range of virulent fungal, bacterial, and viral pathogens. The results showed resistance to both late blight and two powdery mildew species (*Oidium neolycopersici* and *Golovinomyces orontii*). The authors concluded that the *S* gene function of *DND1* is conserved not only in tomato but also in potato [134]. 

Silencing one or more genes via transgene-mediated post-transcriptional gene silencing utilizing RNA interference has led to the emergence of various novel phenotypes [135]. On the other hand, the RNAi construct inserted into *Agrobacterium tumefaciens* aimed at providing durable resistance to *P. infestans* was successfully integrated into the genome of transformed plantlets of the Atlantic variety [136]. Transgenic potato lines (*S.tuberosum* cv. Desirée) were successfully developed to express fused viral coat protein coding sequences from Potato virus X (PVX), Potato virus Y (PVY), and Potato virus S (PVS) as a 600 bp inverted repeat driven by a constitutive 35S promoter [137]. These transgenic lines exhibited 100% resistance to PVX, PVY, and PVS infections compared to untransformed controls, demonstrating the effectiveness of RNA interference (RNAi). 

Conversely, plants employ RNA interference (RNAi) to modulate the expression of pathogen genes encoding effectors, a process known as Host-Induced Gene Silencing (HIGS). HIGS could be used to alter the *S. tuberosum*-*P. infestans* interaction outcome in compatible interactions. In this mechanism, the plant produces double-stranded RNA (dsRNA), targeting a specific gene in the pathogen, leading to the suppression of that gene’s expression [138]. HIGS can limit pathogen growth by targeting genes implicated in the development and/or growth of the pathogen, pathogenesis, or by silencing effector genes that are affecting plant immunity responses [139] (signal transduction, pathogenesis-related proteins, hypersensitive reaction). HIGS can be used by obtaining transgenic plants expressing RNAi constructs, by micro-bombardment, or by spraying. According to Padilla-Roji et al. [139], HIGS has been used mainly for powdery mildews in the pathosystem *Hordeum vulgare*-*Blumeria graminis* f.sp. *hordei* and resulted in a pathogen growth reduction, even in the absence of the gene *Mla10* (*R* gene of barley to powdery mildew). Schaefer et al. [140] silenced three effector genes of *Blumeria graminis* f.sp. *tritici* (*SvrPm3^a1/f1^*, *Bgt-Bcg-6*, and *Bgt-Bcg-7*), simultaneously resulting in the enhanced resistance of wheat plants. HIGS was also successful in reducing disease severity in the pathosystem *Triticum aestivum*-*Puccinia striiformis* f. sp. *tritici* by silencing the effector genes Pst_4 and Pst_5 [141]. 

In the *S. tuberosum-P. infestans* pathosystem, Jahan et al. [142], observed that silencing the PiGPB1 gene resulted in a significant restriction in disease progression. This gene is believed to play a critical role in sporangia formation [143]. When PiGPB1 is silenced, sporangia production is either completely inhibited or results in defective sporangia [142,144]. Sporangia plays a crucial role in disease propagation. Under optimal conditions for pathogen development—such as suitable temperature, water availability, and air humidity—each sporangium produces multiple motile asexual spores, known as zoospores, which infect the plant. Even under suboptimal conditions, sporangia can act as infection propagules and directly initiate disease [145].

Transgene-mediated post-transcriptional gene silencing using RNA interference (RNAi) is a method used to achieve a genetically improved phenotype by inactivating one or more genes [135]. As we understand, RNAi is a fundamental cellular defence mechanism against invading pathogens, but adding in vitro synthesized dsRNA, it can be exploited as a late blight management strategy in potato crops [146]. Also known as spray-induced gene silencing (SIGS), this approach to disease management is an environmentally friendly approach to disease management as it does not leave chemical residues in crops. Additionally, due to its sequence specificity, it only inhibits the target organisms. 

Gene silencing, initiated by double-stranded RNA (dsRNA), has proven to be an invaluable method for identifying and validating gene functions in numerous organisms. In the case of *P. infestans*, these strategies have relied on stable transformation followed by the spontaneous silencing of both the endogenous gene and the transgene [147]. These authors developed the first application of transient gene silencing in *P. infestans*, by delivering in vitro synthesized dsRNA into protoplasts to trigger silencing. After exposure to dsRNA, a reduction in GFP fluorescence was observed in *gfp* dsRNA-treated lines, along with decreased *INF1* production in *inf1* dsRNA-treated lines. Recently, Sundaresha et al. (2022) [148] developed target-specific RNA interference (RNAi)-based molecules, combined with nanoclay carriers (as a carrier for sorbitol dehydrogenase), and the late blight infection was reduced when dsRNA was sprayed on plants. 

Kalyandurg et al. [146] utilized nanoclay combined with double-stranded RNA (dsRNA), targeting several critical genes involved in the pathogenesis of *Phytophthora infestans* or expressed at different stages of its infection cycle: PiGPB1 (sporangia formation), PiOSBP (fungicide target), PiHmp1 (infection establishment), PiCut3 (the carbohydrate-active enzymes cutinase), and PiEndo3 (host tissue penetration and colonization). The results demonstrated that plants treated with the multigene-targeted dsRNA–nanoclay exhibited significantly enhanced disease resistance, showing only 4% disease severity and reduced sporulation compared to plants treated with dsRNA alone. This study validated the dsRNA–nanoclay spray-induced gene silencing (SIGS) method as a proof of concept, suggesting it as a potential alternative to chemical fungicides and transgenic approaches for environmentally friendly and innovative plant protection against late blight.

The dsRNA generated by the bacteria was assessed by Ivanov and his team [149] on potato explants and exhibited a statistically notable decrease in lesions five days post-inoculation compared to the water treatment. dsRNA-based strategies have emerged as promising tools for managing *P. infestans*, the pathogen responsible for late blight disease in potatoes and tomatoes. The efficacity of dsRNA in late blight of tomato was demonstrate by Porwel et al. [150]. When the concentrations of dsRNA were reduced, the spread of infection was limited in leaves, but on petiols, the highest concentration of dsRNA produced the minimum infection. So, dsRNA-based strategies have emerged as promising tools for managing *P. infestans*.

In conclusion, dsRNA-based strategies have emerged as promising tools for managing *P. infestans*, the pathogen responsible for late blight disease in potatoes and tomatoes. When these dsRNA molecules are introduced into the pathogen’s environment, they can trigger a process called RNA interference (RNAi). In RNAi, the dsRNA is recognized by the pathogen’s RNAi machinery, leading to the degradation of the corresponding mRNA molecules and, ultimately, silencing the target genes. So, when the pathogen infects the plant, the expressed dsRNA molecules are taken up by the pathogen, leading to gene silencing and reduced disease development. Also, one of the advantages of dsRNA-based strategies is their specificity. dsRNA molecules can be designed to target specific genes in *Phytophthora infestans*, reducing the environmental impact compared to chemical pesticides. Additionally, dsRNA molecules are biodegradable and do not persist in the environment for extended periods, further enhancing their environmental safety. 

### 3.7. Gene Editing Technologies—CRISPR Cas9 System

CRISPR (Clustered Regularly Interspaced Short Palindromic Repeats) represents a groundbreaking gene editing methodology enabling precise DNA modifications within organisms. It has rapidly become one of the most powerful tools in molecular biology and genetic engineering. CRISPR/Cas9 technology has indeed revolutionized plant biology and agriculture, providing unprecedented opportunities for precise genetic modifications and the development of improved crop varieties.

The CRISPR method is based on a natural defence mechanism found in bacteria, and it has exceeded all expectations in changing plant molecular biology [151]. Bacteria use CRISPR sequences to defend themselves against viruses by storing snippets of viral DNA in their own genomes. CRISPR-Cas is renowned for its robustness, high specificity, and programming capabilities, which enable the precise genetic manipulation of crop species [152].

Cas9 protein, from *Streptococcus pyogenes* (SpCas9), is an enzyme that acts as molecular scissors and is the most popular CRISPR nuclease [153]. It binds to the crRNA (short RNA sequences that are complementary to the target DNA sequence) and guides it to the target DNA sequence. Once there, Cas9 cuts the DNA at the precise location specified by the crRNA [151]. Additionally, it is crucial that the Cas9 protein is situated within the nucleus, where the targeted gene editing processes occur. To create high levels of Cas9 protein, it is common to use a promoter. Examples include the CaMV 35S promoter, the maize ubiquitin promoter [154], the AtU6-26 promoter in *Arabidopsis*, and the OsU6-2 promoter in rice [155].

Advancements in whole-genome sequencing have facilitated the identification of essential gene functions, thereby enhancing the efficiency of CRISPR-Cas9 editing for targeting specific genes. This technology accelerates the development of new germplasm resources with improved agroeconomic traits [156]. Since its initial report a decade ago, the CRISPR-Cas9 system has been successfully used to edit several plant species, including potatoes [155]. 

Potatoes have a tetraploid genome and commonly show a vegetative reproduction mechanism. To generate potato mutants using one-stop engineering processes with programmable nucleases, there are many challenges that need to be overcome [157]. However, in the last decade, a lot of studies have improved economic traits, like yield, quality, biotic stress resistance, and abiotic stress tolerance. Also, studies were conducted to improve the nutritional quality or starch quality of crops, more precisely and accurately using this revolutionary breeding method, known as Clustered Regularly Interspaced Short Palindromic Repeats (CRISPR). 

CRISPR/Cas has proven to be a viable and efficient technology for accelerating potato breeding efforts. Utilizing pathogen-resistant cultivars is anticipated to enhance crop yield and reduce the reliance on fungicides in agriculture. However, the effectiveness of resistance (*R*) genes in potato breeding is limited due to their specificity to particular pathogen races, making them susceptible to being quickly overcome by pathogen evolution [132]. Table 1 presents a collection of studies showcasing the application of this genetic editing method in developing potatoes resistant to *P. infestans.*

In order to develop potato cultivars resistant to late blight in the future, the initial stage of identifying potential susceptibility genes (*S* genes) in potatoes is crucial. Hence, susceptibility genes (*S* genes) play an important role in pathogenesis, and the loss of *S*-gene function can lead to increased resistance [132]. The CRISPR/Cas9 system was employed with two guide RNAs to generate tetra-allelic deletion mutants, which were subsequently evaluated for their resistance to late blight in plants. The functional knockout of specific genes (*StDND1*, *StCHL1*, and *DMG400000582* or *StDMR6-1*) resulted in potatoes with increased resistance against late blight. Also, mutations in the *StDND1* gene had additional effects on plant growth, while mutations in *StDMR6-1* and *StCHL1* did not cause any observable changes in growth phenotype. This indicates their suitability for further agricultural studies. 

CRISPR-Cas9 was used to correct an SNP mutation in the *StCCoAOMT* gene of the late blight-susceptible Russet Burbank (RB) potato genotype. Subsequent metabolic profiling revealed a significant increase in the accumulation of resistance-related metabolites associated with suberization and lignification in the RB genotype [158]. In contrast to Cas9, the expression of catalytically active Cas 12 (from *Lachnospiraceae bacterium*) was used by Ah-Fong et al. [159], because Cas9 was found to be toxic. The authors successfully used Cas12 to achieve editing by employing vectors where the nuclease and its guide RNA were expressed from a single transcript. Additionally, CRISPR-Cas9 was used to edit the elicitor gene *Inf1*, resulting in up to 13% of transformants exhibiting resistance to *P. infestans*. Moon et al. [160] efficiently edited the *StSR4* gene in protoplast cells using the CRISPR-Cas9 system. After introducing the mutation into regenerated potato plants, a maximum efficiency of 34% was observed. Additionally, the elevated salicylic acid (SA) levels in the stsr4 mutant provided greater resistance to *P*. *infestans* compared to the wild type. Bi and his team [161] recently identified a novel susceptibility factor named *StPM1* in *S. tuberosum*. Their study showed that employing CRISPR/Cas9 with two sgRNAs to knockout the gene significantly increased resistance to *Phytophthora*, without adverse effects on potato growth and development. The *NRL1* gene, encoding negative regulators of plant immune signalling and classified within the *S*-gene group, functions as a susceptibility factor, promoting late blight disease [162]. Nourozi et al. [163] employed CRISPR/Cas9 technology with four guide RNAs to target this gene. They obtained 60 transgenic lines regenerated from the Agria cultivar. One mutant line exhibited an approximately 90% reduction in the *StNRL1* expression level, resulting in enhanced resistance to *P. infestans*. Surprisingly, the mutant lines were found to be susceptible to *A. alternata*, suggesting that the *StNRL1* gene plays a role in *P. infestans* infection but confers resistance to *A. alternata*.

**Table 1:** Results obtained by using the CRISPR/Cas genome editing technology for resistance to *P. infestans* in potato.

System Used	Target Gene	Genotype	Main Findings	Reference
CRISPR/Cas 9	*StDND1*, *StCHL1* and *StDMR6-1* (S-genes: Susceptibility genes)	Desirée	gene knockout of *StDMR6-1* and *StCHL1* genes;	[132]
CRISPR/Cas 9	Caffeoyl-CoA Omethyltransferase (*StCCoAOMT*)	Russet Burbank	gene knockout; mutants with late blight resistance;	[158]
CRISPR/Cas 12	*Inf1*	Isolate 1306 and 618	small in-frame mutations resulted in slightly smaller *INF*1 variants.	[159]
CRISPR/Cas 9	*StSR4* gene	Desirée	StSR4 mutants induced salicylic acid (SA) accumulation, improved resistance to *P. infestans*	[160]
CRISPR/Cas 9	*StPM1* gene	Phureja S15-65 clone	knockout the gene	[161]
CRISPR/Cas 9	*StNRL1* gene	Agria	silencing *StNRL1* enhances resistance to *P. infestans.*	[163]

The CRISPR/Cas system, being the predominant genome editing technique, can accelerate plant breeding by enabling rapid, precise, and predictable genome modifications, resulting in significant advancements in crop breeding [164]. Also, potato breeding has already seen significant gains from the CRISPR/Cas system, and we anticipate many more exciting developments will follow. 

### 3.8. Gene Pyramiding

A major issue in late blight resistance breeding is durability. The conventional strategy is the introgression of a single *Rpi* gene. With a pathogen evolving so fast, like *P. infestans*, this resistance is generally quickly overcome [165]. A solution to enhance resistance durability is to make it harder for the pathogen to break it. One of the strategies proposed is gene pyramiding (GP). GP or gene stacking is the accumulation of two or more *R* genes on the same individual plant. When a gene pyramiding program is completed, a genotype including every target gene is produced [166]. And this is supposed to increase the resistance durability toward diseases [167]. In addition to the higher resistance against pests and pathogens, these gene pyramids also increase the resistance stability over time, reducing the probability of simultaneously overcoming all genes, especially when broad-spectrum *R* genes are used [168]. This method has certain objectives: (1) the increasing trait performance following the combination of complementary genes; (2) the durability increases; and (3) introgressing genes from different sources to correct the deficits [166]. 

Cultivars with gene pyramids could be created using the conventional breeding strategies, such as Pedigree, Backcross, and Recurrent selection [166]. Alternatively, biotechnological tools like somatic hybridization and DNA recombinant technologies can also be employed. Both approaches can significantly benefit from marker-assisted selection [60,165].

Resistance GP is performed by introducing and accumulating multiple *R* defence genes that confer resistance against different pathogen races [168]. Other than the use of *R* genes, which are specialized in recognizing the pathogen, other types of genes could also be used, for instance, genes coding for the pathogenesis-related proteins (chitinase, glucanase, etc.) and genes coding for enzymes implicated in the biosynthesis of secondary metabolites. However, the primary focus of breeding for resistance against *Phytophthora infestans* in potatoes has been on the introgression of resistance genes (*R*) [169]. 

Many genotypes with highly effective and durable resistance have pyramided *Rpi* genes. For instance, Sarpo Mira has six *Rpi* genes, from which five genes, respectively, *R3a*, *R3b*, *R4*, and the newly identified *Rpi-Smira1*, control the qualitative resistance [51]. The quantitative resistance was determined by the *Rpi-Smira2* gene. Kim et al. [74] demonstrated that in the genotypes MaR8 and MaR9, at least four genes of types *R3a*, *R3b*, *R4*, and *R8* are present, as well as seven of types *R1*, *Rpi-abpt1*, *R3a*, *R3b*, *R4*, *R8*, *R9*, respectively. The Fortuna variety has two pyramided genes, namely *Rpi-blb1* and *Rpi-blb2* [170]. The Bzura cultivar harbours two *Rpi* genes: *R2-like* and another unknown *Rpi* gene [171]. Two resistance genes, *Rpi-mcd1* and *Rpi-ber*, introgressed from the wild potato species *S. microdontum* and *S. berthaultii*, respectively, were combined in a diploid *S. tuberosum* by Tan and his team [172]. The results suggest that pyramiding can result in an additive effect of the individual genes on the level of resistance.

The non-host resistance of pepper to *P. infestans* is due to *Rpi* gene pyramids. Oh et al. [173] found that pepper (*Capsicum annuum*) non-host resistance to late blight is based on a pyramid of *Rpi* genes that are very close to potato ones, able to recognize *P. infestans* effectors, such as Avr2, Avrblb1, Avrblb2, and Avrvnt1.

Even defeated *Rpi* genes can enhance potato resistance [168,174]. Also, Taoutaou et al. [175] showed that gene pyramiding also broadens the resistance spectrum of potato. Recently, Taoutaou et al. [168] showed that pyramids of the defeated *Rpi* gene delay the disease installation and reduce the quantity of sporangia produced by the pathogen; the area of the lesion was also reduced significantly in the genotypes harbouring four *Rpi* genes. Also, these genotypes produce more phenolic compounds [176].

In addition to the *Rpi* genes, potato and other plants are equipped with pattern recognition receptors (PRRs) that can detect apoplastic effectors or pathogen-associated molecular patterns (PAMPs) on the cell surface [169]. Potato genotypes overexpressing PRR are less affected by *P. infestans* attacks when compared to the single transformants. These findings suggest that pyramiding PRRs can also give additional increased resistance against pathogens like *P. infestans* [169].

However, breaking resistance based on pyramids is harder for the pathogen, though, at least theoretically, it happened. This is the case for the cultivar Vertifolia. It is a potato cultivar harbouring two *Rpi* genes (*R3* and *R4*) [177]. There is a term for it: the Vertifolia effect. It is defined by the American Society of Phytopathology as follows: “*the loss of general (durable, horizontal, host-nonspecific) resistance in a cultivar after several generations of selection during which a major gene confers resistance to the dominant race or biotype of the pathogen; first observed in the potato cultivar Vertifolia with late blight resistance*” (APS: https://www.apsnet.org/edcenter/resources/illglossary/Pages/S-V.aspx, accessed on 16 May 2024). 

Drawing from the pathogen’s ability to overcome resistance, even cultivars with gene pyramids are susceptible to breakdown. To avoid this risk, implementing a strategic deployment of resistance, including pyramided broad-spectrum *R* genes, may be essential. 

Bradshaw [178] highlighted the evident necessity for developing new potato cultivars, despite the abundance of existing varieties. Emphasizing the importance of incorporating inherent resistance to diseases and pests, these novel cultivars are crucial for sustaining agricultural productivity. Being the most dangerous pathogen on potato, breeding for *P. infestans* resistance is a major priority. 

As is known, *P. infestans* is a highly evolving pathogen. It has the largest and most complex genome of the chromalveolates (240 megabases), with 74% of it as proliferative repetitive DNA [179]. In Canada, Babarinde et al. [180] found that 25 new genotypes are emerging, as well as the already existing US11, US17, US8, and US23. Also, to illustrate this pathogen’s high evolutive ability, Shen et al. [181] analysed the sequence of only one effector (Avr1) of 111 Chinese *P. infestans* isolates, during a short period (2010–2011), and found that nearly 80% of the isolates have undergone a point mutation. In another study, Yang et al. [182] found that the effector gene Pi02860 has undergone moderate genetic alteration, mainly by point mutation, but resulted in a modification in pathogen fitness. As mentioned above, *P. infestans* harbours more than 500 RxRL effectors, and this is only one type of effector. When considering the potential effectors and their ability to evolve, and its consequence on pathogen fitness and pathogenicity, this pathogen could be uncontrollable. 

This property make resistance breeding a real challenge for breeders due to the durability issue of resistance. In addition to pyramiding, another strategy is to target a vital process in the pathogen. For instance, Yang et al. [183] transformed potato by expressing secreted *A. thaliana* phosphatidylinositol-4-phosphate 5-kinase 1 (AtPIP5K1) (it phosphorylates *Phytophthora*’s phosphatidylinositol 3-phosphate (PI3P) to PI(3,4)P_2_) and, as a result, obtained potato plants with enhanced catabolism of PI3P, promoting infection. The obtained plants were significantly more resistant. Such innovative strategies, like those cited above, need a priory much better understanding of pathogen biology and pathogenicity. In the same way, effectoromics made it easier to mine *R* genes [184]. 

Conventionally, late blight breeding is based on the introgression of *R* genes. The adoption of *R* gene pyramiding boosted the use of these genes [7]. The major issue with *R* gene-based breeding is in the function. Even though R proteins are highly specific in their function, basically, they play the same role as PRR (surveillance of pathogen effectors) [185]. And they have a major parameter to take into consideration: elicitors (detected by PRR) do not evolve as quickly as effectors (detected by R protein). 

The discovery of iRNA and its implication in plant–pathogen interactions open new exciting opportunities for resistance breeding (Hou and Ma., 2020). The use of HIGS could be a much better option, when a critical and vital gene is targeted. It has been used successfully against viruses [186,187]. The main issue for the use of HIGS in Europe is the way that it is obtained via genetic transformation. Currently, we know that plants, at least *Arabidopsis*, produce iRNA against several pathogens, including *Botrytis cinerea*, *Verticillium dahliae*, and even *Phytophthora capsici* [188,189,190]. More research is needed to identify naturally occurring potato HIGS that could be exploited to enhance resistance to *P. infestans*. 

Potato susceptibility, *P. infestans* biology, and pathogenicity are key factors in modern breeding. For a pathogen like *P. infestans*, a combinatorial biotechnological approach is needed. Several strategies and tools should be considered to create a potato genotype with a better chance to stand out against this pathogen, for instance, enhancing pathogen detection and recognition via gene pyramiding and also PRR, combined with susceptibility gene silencing. Also, better resistance deployment is needed to optimize the durability and effectiveness of the already available resistance. 

## 4. Conclusions

In this study, we delved into the technologies and approaches used to enhance potato resistance to late blight, the major disease in potato. We did not discuss the classical approach of crossing (natural hybridization), but it is still a viable technique, especially when combined with the technologies and approaches cited here, and this should certainly be developed in the future. Even the most basic approaches could benefit from modern technologies. The huge step was including the recent scientific advances in pathogen biology and pathogenicity by using effectors to search for *R* genes (effectoromics). As we have seen, genome editing has great potential, especially using the CRISPR gene editing system, but RNAi is also a valuable technique due to its ability to precisely target genes (important genes in pathogen biology and/or pathogenicity) to be silenced. Priority should be given to tools that do not leave traces of exogenous DNA to simplify regulatory processes. Screening germplasms is crucial for identifying desirable traits present in these wild species that can be introduced into cultivated potato varieties through breeding (introgression). On the other hand, targeting susceptibility genes by gene silencing, gene editing will make it harder for the pathogen to recognize, colonize, and cause disease in potatoes. Further work for the identification of these genes is necessary. Exploiting the HIGS is also a promising approach. This approach also requires a pathogen-informed breeding strategy. dsRNA-based strategies have emerged as promising tools for managing *P. infestans*. These approaches could become very efficient, if integrated in the process of resistance breeding, as part of a pathogen-informed breeding strategy. Durable resistance breeding against this pathogen will continue to be a challenge, at least in the near future. The development of new technologies enabling researchers and breeders to target evolutive costly traits for the pathogen will be of great help. Also, the introgression of *R* genes that are able to recognize conserved effectors in *Phytophthora* species, *Perenosporaceae* family, or maybe in the *Oomycete phylum* (wide-spectrum *R* genes), alone or in combination with other *R* genes, and/or with other genes involved in the potato immune system should contribute to enhanced resistance durability. Maybe for this pathogen, the best method is a multi-approach strategy, where several tools are combined. 

## Data Availability

Not applicable.

## References

[1] Schmey T., Tominello-Ramirez C.S., Brune C., Stam R. (2024). Alternaria Diseases on Potato and Tomato. Mol. Plant Pathol..

[2] Azil N., Stefańczyk E., Sobkowiak S., Chihat S., Boureghda H., Śliwka J. (2021). Identification and Pathogenicity of *Fusarium* spp. Associated with Tuber Dry Rot and Wilt of Potato in Algeria. Eur. J. Plant Pathol..

[3] Valkonen J.P., Vreugdenhil D., Bradshaw J., Gebhardt C., Govers F., MacKerron D.K.L., Taylor M.A., Ross H.A. (2007). Potato viruses: Economical losses and biotechnological potential. Potato Biology and Biotechnology: Advances and Perspectives.

[4] Termorshuizen A.J., Vreugdenhil D., Bradshaw J., Gebhardt C., Govers F., MacKerron D.K.L., Taylor M.A., Ross H.A. (2007). Fungal and Fungus-Like Pathogens of Potato. Potato Biology and Biotechnology: Advances and Perspectives.

[5] van der Wolf J.M., de Boer S.H., Vreugdenhil D., Bradshaw J., Gebhardt C., Govers F., MacKerron D.K.L., Taylor M.A., Ross H.A. (2007). Bacterial pathogens of potato. Potato Biology and Biotechnology: Advances and Perspectives.

[6] Agrios G.N. (2008). Plant Pathology.

[7] Ivanov A.A., Ukladov E.O., Golubeva T.S. (2021). *Phytophthora infestans*: An Overview of Methods and Attempts to Combat Late Blight. J. Fungi.

[8] Fry W.E., Birch P.R.J., Judelson H.S., Grünwald N.J., Danies G., Everts K.L., Gevens A.J., Gugino B.K., Johnson D.A., Johnson S.B. (2015). Five Reasons to Consider *Phytophthora infestans* a Reemerging Pathogen. Phytopathology.

[9] Taoutaou A., Berindean I.V., Botez C., Beninal L., Pamfil D. (2024). The Dynamics of Populations of *Phytophthora infestans* (Mont.) De Bary, In the Central Eastern Europe, Part 1: Up To 2010. RAR.

[10] Hohl H.R., Iselin K. (1984). Strains of *Phytophthora infestans* from Switzerland with A2 Mating Type Behaviour. Trans. Br. Mycol. Soc..

[11] Cooke D.E.L., Cano L.M., Raffaele S., Bain R.A., Cooke L.R., Etherington G.J., Deahl K.L., Farrer R.A., Gilroy E.M., Goss E.M. (2012). Genome Analyses of an Aggressive and Invasive Lineage of the Irish Potato Famine Pathogen. PLoS Pathog..

[12] Fry W.E., Goodwin S.B., Dyer A.T., Matuszak J.M., Drenth A., Tooley P.W., Sujkowski L.S., Koh Y.J., Cohen B.A., Spielman L.J. (1993). Historical and Recent Migrations of *Phytophthora infestans*: Chronology, Pathways and Implications. Plant Dis..

[13] Machida-Hirano R. (2015). Diversity of Potato Genetic Resources. Breed. Sci..

[14] Gopal J., Khurana S.M. (2006). Handbook of Potato Production, Improvement, and Postharvest Management.

[15] Bradshaw J.E., Bryan G.J., Ramsay G. (2006). Genetic Resources (Including Wild and Cultivated Solanum Species) and Progress in Their Utilisation in Potato Breeding. Potato Res..

[16] Spooner D.M., Ghislain M., Simon R., Jansky S.H., Gavrilenko T. (2014). Systematics, Diversity, Genetics, and Evolution of Wild and Cultivated Potatoes. Bot. Rev..

[17] Paluchowska P., Śliwka J., Yin Z. (2022). Late Blight Resistance Genes in Potato Breeding. Planta.

[18] Malcolmson J.F., Black W. (1966). New R Genes in *Solanum demissum* Lindl. And Their Complementary Races of *Phytophthora infestans* (Mont.) de Bary. Euphytica.

[19] Black W., Mastenbroek C., Mills W.R., Peterson L.C. (1953). A Proposal for an International Nomenclature of Races of *Phytophthora infestans* and of Genes Controlling Immunity in Solanum Demissum Derivatives. Euphytica.

[20] Vossen J.H., van Arkel G., Bergervoet M., Jo K.-R., Jacobsen E., Visser R.G.F. (2016). The *Solanum demissum R8* Late Blight Resistance Gene Is an Sw-5 Homologue That Has Been Deployed Worldwide in Late Blight Resistant Varieties. Appl. Genet..

[21] Park T.-H., Foster S., Brigneti G., Jones J. (2009). Two Distinct Potato Late Blight Resistance Genes from *Solanum berthaultii* Are Located on Chromosome 10. Euphytica.

[22] Monino-Lopez D., Nijenhuis M., Kodde L., Kamoun S., Salehian H., Schentsnyi K., Stam R., Lokossou A., Abd-El-Haliem A., Visser R.G.F. (2021). Allelic Variants of the NLR Protein Rpi-Chc1 Differentially Recognize Members of the *Phytophthora infestans* PexRD12/31 Effector Superfamily through the Leucine-Rich Repeat Domain. Plant J..

[23] van der Vossen E., Sikkema A., Hekkert B.T., Gros J., Stevens P., Muskens M., Wouters D., Pereira A., Stiekema W., Allefs S. (2003). An Ancient R Gene from the Wild Potato Species *Solanum bulbocastanum* Confers Broad-Spectrum Resistance to *Phytophthora infestans* in Cultivated Potato and Tomato. Plant J..

[24] Park T.-H., Gros J., Sikkema A., Vleeshouwers V.G.A.A., Muskens M., Allefs S., Jacobsen E., Visser R.G.F., van der Vossen E.A.G. (2005). The Late Blight Resistance Locus *Rpi-Bib3* from *Solanum bulbocastanum* Belongs to a Major Late Blight *R* Gene Cluster on Chromosome 4 of Potato. Mol. Plant Microbe Interact.

[25] Li J., Kaur A., Harrower B., Armstrong M., Dou D., Wang X., Hein I. (2023). Identification and Mapping of *Rpi-Blb4* in Diploid Wild Potato Species *Solanum bulbocastanum*. Crop J..

[26] Vleeshouwers V.G.A.A., Rietman H., Krenek P., Champouret N., Young C., Oh S.-K., Wang M., Bouwmeester K., Vosman B., Visser R.G.F. (2008). Effector Genomics Accelerates Discovery and Functional Profiling of Potato Disease Resistance and *Phytophthora infestans* Avirulence Genes. PLoS ONE.

[27] Wang M., Allefs S., Van Den Berg R.G., Vleeshouwers V.G.A.A., Van Der Vossen E.A.G., Vosman B. (2008). Allele Mining in Solanum: Conserved Homologues of *Rpi-Blb1* Are Identified in *Solanum stoloniferum*. Appl. Genet..

[28] Foster S.J., Park T.-H., Pel M., Brigneti G., Sliwka J., Jagger L., van der Vossen E., Jones J.D.G. (2009). *Rpi-Vnt1.1*, a Tm-2(2) Homolog from *Solanum venturii*, Confers Resistance to Potato Late Blight. Mol. Plant Microbe Interact.

[29] Karki H.S., Jansky S.H., Halterman D.A. (2021). Screening of Wild Potatoes Identifies New Sources of Late Blight Resistance. Plant Dis..

[30] Duan Y., Duan S., Xu J., Zheng J., Hu J., Li X., Li B., Li G., Jin L. (2021). Late Blight Resistance Evaluation and Genome-Wide Assessment of Genetic Diversity in Wild and Cultivated Potato Species. Front. Plant Sci..

[31] Rogozina E.V., Beketova M.P., Muratova O.A., Kuznetsova M.A., Khavkin E.E. (2021). Stacking Resistance Genes in Multiparental Interspecific Potato Hybrids to Anticipate Late Blight Outbreaks. Agronomy.

[32] Cooke D.E.L., Lees A.K. (2004). Markers, Old and New, for Examining *Phytophthora infestans* Diversity. Plant Pathol..

[33] Manuela H. (2018). The Influence of Cultivar Resistance at the Onset of Potato Late Blight. J. Hortic. For. Biotechnol..

[34] Hermeziu M., Hermeziu R. (2019). A New Romanian Potato Variety, Cezarina and its Specific Technology. Agricultura.

[35] Nahirñak V., Almasia N.I., González M.N., Massa G.A., Décima Oneto C.A., Feingold S.E., Hopp H.E., Vazquez Rovere C. (2022). State of the Art of Genetic Engineering in Potato: From the First Report to Its Future Potential. Front. Plant Sci..

[36] Flor H.H. (1971). Current Status of the Gene-For-Gene Concept. Annu. Rev. Phytopathol..

[37] Zipfel C. (2014). Plant Pattern-Recognition Receptors. Trends Immunol..

[38] Roudaire T., Héloir M.-C., Wendehenne D., Zadoroznyj A., Dubrez L., Poinssot B. (2021). Cross Kingdom Immunity: The Role of Immune Receptors and Downstream Signaling in Animal and Plant Cell Death. Front. Immunol..

[39] Bezerra-Neto J.P., Araújo F.C., Ferreira-Neto J.R.C., Silva R.L.O., Borges A.N.C., Matos M.K.S., Silva J.B., Silva M.D., Kido E.A., Benko-Iseppon A.M., Poltronieri P., Hong Y. (2020). Chapter 4—NBS-LRR Genes—Plant Health Sentinels: Structure, Roles, Evolution and Biotechnological Applications. Applied Plant Biotechnology for Improving Resistance to Biotic Stress.

[40] Rodewald J., Trognitz B. (2013). Solanum Resistance Genes against *Phytophthora infestans* and Their Corresponding Avirulence Genes. Mol. Plant Pathol..

[41] Song J., Bradeen J.M., Naess S.K., Raasch J.A., Wielgus S.M., Haberlach G.T., Liu J., Kuang H., Austin-Phillips S., Buell C.R. (2003). Gene *RB* Cloned from *Solanum bulbocastanum* Confers Broad Spectrum Resistance to Potato Late Blight. Proc. Natl. Acad. Sci. USA.

[42] van der Vossen E.A.G., Gros J., Sikkema A., Muskens M., Wouters D., Wolters P., Pereira A., Allefs S. (2005). The *Rpi-Blb2* Gene from *Solanum bulbocastanum* Is an *Mi-1* Gene Homolog Conferring Broad-Spectrum Late Blight Resistance in Potato. Plant J..

[43] Lin X., Olave-Achury A., Heal R., Pais M., Witek K., Ahn H.-K., Zhao H., Bhanvadia S., Karki H.S., Song T. (2022). A Potato Late Blight Resistance Gene Protects against Multiple *Phytophthora* Species by Recognizing a Broadly Conserved RXLR-WY Effector. Mol. Plant.

[44] van Schie C.C.N., Takken F.L.W. (2014). Susceptibility Genes 101: How to Be a Good Host. Annu. Rev. Phytopathol..

[45] Kamoun S. (2006). A Catalogue of the Effector Secretome of Plant Pathogenic Oomycetes. Annu. Rev. Phytopathol..

[46] Du J., Vleeshouwers V.G.A.A., Birch P., Jones J.T., Bos J.I.B. (2014). The Do’s and Don’ts of Effectoromics. Plant-Pathogen Interactions: Methods and Protocols.

[47] Vleeshouwers V.G.A.A., Raffaele S., Vossen J.H., Champouret N., Oliva R., Segretin M.E., Rietman H., Cano L.M., Lokossou A., Kessel G. (2011). Understanding and Exploiting Late Blight Resistance in the Age of Effectors. Annu. Rev. Phytopathol..

[48] Vleeshouwers V.G.A.A., Oliver R.P. (2014). Effectors as Tools in Disease Resistance Breeding against Biotrophic, Hemibiotrophic, and Necrotrophic Plant Pathogens. MPMI.

[49] Domazakis E., Lin X., Aguilera-Galvez C., Wouters D., Bijsterbosch G., Wolters P.J., Vleeshouwers V.G.A.A., Shan L., He P. (2017). Effectoromics-Based Identification of Cell Surface Receptors in Potato. Plant Pattern Recognition Receptors: Methods and Protocols.

[50] Van Weymers P.S.M., Baker K., Chen X., Harrower B., Cooke D.E.L., Gilroy E.M., Birch P.R.J., Thilliez G.J.A., Lees A.K., Lynott J.S. (2016). Utilizing “Omic” Technologies to Identify and Prioritize Novel Sources of Resistance to the Oomycete Pathogen *Phytophthora infestans* in Potato Germplasm Collections. Front. Plant Sci..

[51] Rietman H., Bijsterbosch G., Cano L.M., Lee H.-R., Vossen J.H., Jacobsen E., Visser R.G.F., Kamoun S., Vleeshouwers V.G.A.A. (2012). Qualitative and Quantitative Late Blight Resistance in the Potato Cultivar Sarpo Mira Is Determined by the Perception of Five Distinct RXLR Effectors. MPMI.

[52] Giesbers A.K.J., Pelgrom A.J.E., Visser R.G.F., Niks R.E., Van den Ackerveken G., Jeuken M.J.W. (2017). Effector-Mediated Discovery of a Novel Resistance Gene against *Bremia lactucae* in a Nonhost Lettuce Species. New Phytol..

[53] Collard B.C.Y., Mackill D.J. (2008). Marker-Assisted Selection: An Approach for Precision Plant Breeding in the Twenty-First Century. Philos. Trans. R. Soc. Lond. B Biol. Sci..

[54] Hasan N., Choudhary S., Naaz N., Sharma N., Laskar R.A. (2021). Recent Advancements in Molecular Marker-Assisted Selection and Applications in Plant Breeding Programmes. J. Genet. Eng. Biotechnol..

[55] Ahmar S., Gill R.A., Jung K.-H., Faheem A., Qasim M.U., Mubeen M., Zhou W. (2020). Conventional and Molecular Techniques from Simple Breeding to Speed Breeding in Crop Plants: Recent Advances and Future Outlook. Int. J. Mol. Sci..

[56] Varshney R.K., Graner A., Sorrells M.E. (2005). Genomics-Assisted Breeding for Crop Improvement. Trends Plant Sci..

[57] Rosa P.M., de Campos T., de Sousa A.C.B., Sforça D.A., Torres G.A.M., de Souza A.P. (2010). Potato Cultivar Identification Using Molecular Markers. Pesq. Agropec. Bras..

[58] Rocha E.A., Paiva L.V., Carvalho H.H.D., Guimarães C.T. (2010). Molecular Characterization and Genetic Diversity of Potato Cultivars Using SSR and RAPD Markers. Crop. Breed. Appl. Biotechnol..

[59] Verma V.K., Kumar A., Rymbai H., Talang H., Devi M.B., Baiswar P., Hazarika S. (2023). Genetic Diversity and Stability Analysis of Sweet Potato Accessions of North-Eastern India Grown under the Mid-Hill Conditions of Meghalaya. Plant Genet. Resour..

[60] Islam S., Li J., Rahman M.A., Xie F., Song B., Nie B. (2024). Resistance to Biotic and Abiotic Stress in Potato: The Origin of the Genes and Corresponding Molecular Markers. Phytopathol. Res..

[61] Tiwari J.K., Siddappa S., Singh B.P., Kaushik S.K., Chakrabarti S.K., Bhardwaj V., Chandel P. (2013). Molecular Markers for Late Blight Resistance Breeding of Potato: An Update. Plant Breed..

[62] Taoutaou A., Berindean I.V., Csete E., Pamfil D., Botez C. (2022). Development of a Molecular Marker for the Resistance Gene *R11* of Potato to Late Blight. RAR.

[63] Yousaf M.F., Demirel U., Naeem M., Çalışkan M.E. (2021). Association Mapping Reveals Novel Genomic Regions Controlling Some Root and Stolon Traits in Tetraploid Potato (*Solanum tuberosum* L.). 3 Biotech.

[64] Sinha D., Maurya A.K., Abdi G., Majeed M., Agarwal R., Mukherjee R., Ganguly S., Aziz R., Bhatia M., Majgaonkar A. (2023). Integrated Genomic Selection for Accelerating Breeding Programs of Climate-Smart Cereals. Genes.

[65] Ghislain M. (1997). Biotechnology and the Potato: Applications for the Developing World.

[66] Keijzer P., van Bueren E.T.L., Engelen C.J.M., Hutten R.C.B. (2022). Breeding Late Blight Resistant Potatoes for Organic Farming—A Collaborative Model of Participatory Plant Breeding: The Bioimpuls Project. Potato Res..

[67] Caruana B.M., Pembleton L.W., Constable F., Rodoni B., Slater A.T., Cogan N.O.I. (2019). Validation of Genotyping by Sequencing Using Transcriptomics for Diversity and Application of Genomic Selection in Tetraploid Potato. Front. Plant Sci..

[68] Slater A.T., Schultz L., Lombardi M., Rodoni B.C., Bottcher C., Cogan N.O.I., Forster J.W. (2020). Screening for Resistance to PVY in Australian Potato Germplasm. Genes.

[69] Gebhardt C., Ballvora A., Walkemeier B., Oberhagemann P., Schüler K. (2004). Assessing Genetic Potential in Germplasm Collections of Crop Plants by Marker-Trait Association: A Case Study for Potatoes with Quantitative Variation of Resistance to Late Blight and Maturity Type. Mol. Breed..

[70] Madhumati B. (2014). Potential and Application of Molecular Markers Techniques for Plant Genome Analysis. Int. J. Pure App. Biosci..

[71] Leal S.M. (2001). Genetics and Analysis of Quantitative Traits. Am. J. Hum. Genet..

[72] Kiran U., Khan S., Mirza K.J., Ram M., Abdin M.Z. (2010). SCAR Markers: A Potential Tool for Authentication of Herbal Drugs. Fitoterapia.

[73] Michaels S.D., Amasino R.M. (1998). A Robust Method for Detecting Single-Nucleotide Changes as Polymorphic Markers by PCR. Plant J..

[74] Kim H.-J., Lee H.-R., Jo K.-R., Mortazavian S.M.M., Huigen D.J., Evenhuis B., Kessel G., Visser R.G.F., Jacobsen E., Vossen J.H. (2012). Broad Spectrum Late Blight Resistance in Potato Differential Set Plants MaR8 and MaR9 Is Conferred by Multiple Stacked R Genes. Appl. Genet..

[75] Sokolova E.A., Fadina O.A., Khavkin E.E., Rogozina E.V., Jones R.W., Deahl K.L. Structural Homologues of CC-NBS-LRR Genes for Potato Late Blight Resistance in Wild Solanum Species. Proceedings of the Fourteenth Euroblight Workshop.

[76] Rietman H. (2011). Putting the Phytophthora infestans Genome Sequence at Work: Multiple Novel Avirulence and Potato Resistance Gene Candidates Revealed.

[77] Chen S., Borza T., Byun B., Coffin R., Coffin J., Peters R., Wang-Pruski G. (2017). DNA Markers for Selection of Late Blight Resistant Potato Breeding Lines. Am. J. Plant Sci..

[78] Islam S., Eusufzai T., Ansarey F., Hasan M., Nahiyan A. (2022). A Breeding Approach to Enhance Late Blight Resistance in Potato. J. Hortic. Sci. Biotechnol..

[79] Jacobs M.M.J., Vosman B., Vleeshouwers V.G.A.A., Visser R.G.F., Henken B., van den Berg R.G. (2010). A Novel Approach to Locate *Phytophthora infestans* Resistance Genes on the Potato Genetic Map. Appl. Genet..

[80] Wickramasinghe W.M.D.K., Qu X.S., Costanzo S., Haynes K.G., Christ B.J. (2009). Development of PCR-Based Markers Linked to Quantitative Resistance to Late Blight in a Diploid Hybrid Potato Population of *Solanum phureja* × *S. stenotomum*. Am. J. Potato Res..

[81] Syverson R.L., Bradeen J.M. (2011). A Novel Class of Simple PCR Markers with SNP-Level Sensitivity for Mapping and Haplotype Characterization in *Solanum* Species. Am. J. Pot. Res..

[82] Rafalski A. (2002). Applications of Single Nucleotide Polymorphisms in Crop Genetics. Curr. Opin. Plant Biol..

[83] Wang F., Zou M., Zhao L., Xia Z., Wang J. (2021). Genome-Wide Association Mapping of Late Blight Tolerance Trait in Potato (*Solanum tuberosum* L.). Front. Genet..

[84] Sood S., Bhardwaj V., Bairwa A., Dalamu, Sharma S., Sharma A.K., Kumar A., Lal M., Kumar V. (2023). Genome-Wide Association Mapping and Genomic Prediction for Late Blight and Potato Cyst Nematode Resistance in Potato (*Solanum tuberosum* L.). Front. Plant Sci..

[85] Ortega F., Lopez-Vizcon C. (2012). Application of Molecular Marker-Assisted Selection (MAS) for Disease Resistance in a Practical Potato Breeding Programme. Potato Res..

[86] Xiao Y., Liu H., Wu L., Warburton M., Yan J. (2017). Genome-Wide Association Studies in Maize: Praise and Stargaze. Mol. Plant.

[87] Alqudah A.M., Sallam A., Stephen Baenziger P., Börner A. (2020). GWAS: Fast-Forwarding Gene Identification and Characterization in Temperate Cereals: Lessons from Barley—A Review. J. Adv. Res..

[88] Glowinski A., Flint-Garcia S., Bennetzen J., Flint-Garcia S., Hirsch C., Tuberosa R. (2018). Germplasm Resources for Mapping Quantitative Traits in Maize. The Maize Genome.

[89] Pradhan S.K., Barik S.R., Sahoo A., Mohapatra S., Nayak D.K., Mahender A., Meher J., Anandan A., Pandit E. (2016). Population Structure, Genetic Diversity and Molecular Marker-Trait Association Analysis for High Temperature Stress Tolerance in Rice. PLoS ONE.

[90] Zhang S., Chen X., Lu C., Ye J., Zou M., Lu K., Feng S., Pei J., Liu C., Zhou X. (2018). Genome-Wide Association Studies of 11 Agronomic Traits in Cassava (*Manihot esculenta* Crantz). Front. Plant Sci..

[91] Hindu V., Palacios-Rojas N., Babu R., Suwarno W.B., Rashid Z., Usha R., Saykhedkar G.R., Nair S.K. (2018). Identification and Validation of Genomic Regions Influencing Kernel Zinc and Iron in Maize. Appl. Genet..

[92] Flint-Garcia S.A., Jampatong C., Darrah L.L., McMullen M.D. (2003). Quantitative Trait Locus Analysis of Stalk Strength in Four Maize Populations. Crop Sci..

[93] Vos P.G., Paulo M.J., Voorrips R.E., Visser R.G.F., Van Eck H.J., Van Eeuwijk F.A. (2017). Evaluation of LD Decay and Various LD-Decay Estimators in Simulated and SNP-Array Data of Tetraploid Potato. Appl. Genet..

[94] Sharma S.K., MacKenzie K., McLean K., Dale F., Daniels S., Bryan G.J. (2018). Linkage Disequilibrium and Evaluation of Genome-Wide Association Mapping Models in Tetraploid Potato. G3 Genes|Genomes|Genet..

[95] Rida S., Maafi O., López-Malvar A., Revilla P., Riache M., Djemel A. (2021). Genetics of Germination and Seedling Traits under Drought Stress in a MAGIC Population of Maize. Plants.

[96] Yi Q., Malvar R.A., Álvarez-Iglesias L., Ordás B., Revilla P. (2020). Dissecting the Genetics of Cold Tolerance in a Multiparental Maize Population. Appl. Genet..

[97] Revilla P., Rodríguez V.M., Ordás A., Rincent R., Charcosset A., Giauffret C., Melchinger A.E., Schön C.-C., Bauer E., Altmann T. (2016). Association Mapping for Cold Tolerance in Two Large Maize Inbred Panels. BMC Plant Biol..

[98] Jiménez-Galindo J.C., Malvar R.A., Butrón A., Santiago R., Samayoa L.F., Caicedo M., Ordás B. (2019). Mapping of Resistance to Corn Borers in a MAGIC Population of Maize. BMC Plant Biol..

[99] Stange M., Utz H.F., Schrag T.A., Melchinger A.E., Würschum T. (2013). High-Density Genotyping: An Overkill for QTL Mapping? Lessons Learned from a Case Study in Maize and Simulations. Appl. Genet..

[100] Simko I., Jansky S., Stephenson S., Spooner D., Vreugdenhil D., Bradshaw J., Gebhardt C., Govers F., MacKerron D.K.L., Taylor M.A., Ross H.A. (2007). Genetics of Resistance to Pests and Disease. Potato Biology and Biotechnology: Advances and Perspectives.

[101] Lindqvist-Kreuze H., De Boeck B., Unger P., Gemenet D., Li X., Pan Z., Sui Q., Qin J., Woldegjorgis G., Negash K. (2021). Global Multi-Environment Resistance QTL for Foliar Late Blight Resistance in Tetraploid Potato with Tropical Adaptation. G3 Genes|Genomes|Genet..

[102] Rosyara U.R., De Jong W.S., Douches D.S., Endelman J.B. (2016). Software for Genome-Wide Association Studies in Autopolyploids and Its Application to Potato. Plant Genome.

[103] Hamilton J.P., Hansey C.N., Whitty B.R., Stoffel K., Massa A.N., Van Deynze A., De Jong W.S., Douches D.S., Buell C.R. (2011). Single Nucleotide Polymorphism Discovery in Elite North American Potato Germplasm. BMC Genom..

[104] Vos P.G., Uitdewilligen J.G.A.M.L., Voorrips R.E., Visser R.G.F., Van Eck H.J. (2015). Development and Analysis of a 20K SNP Array for Potato (*Solanum tuberosum*): An Insight into the Breeding History. Appl. Genet..

[105] Pham G.M., Hamilton J.P., Wood J.C., Burke J.T., Zhao H., Vaillancourt B., Ou S., Jiang J., Buell C.R. (2020). Construction of a Chromosome-Scale Long-Read Reference Genome Assembly for Potato. GigaScience.

[106] Lindqvist-Kreuze H., Gastelo M., Perez W., Forbes G.A., De Koeyer D., Bonierbale M. (2014). Phenotypic Stability and Genome-Wide Association Study of Late Blight Resistance in Potato Genotypes Adapted to the Tropical Highlands. Phytopathology.

[107] Pel M.A., Foster S.J., Park T.-H., Rietman H., Van Arkel G., Jones J.D.G., Van Eck H.J., Jacobsen E., Visser R.G.F., Van Der Vossen E.A.G. (2009). Mapping and Cloning of Late Blight Resistance Genes from *Solanum Venturii* Using an Interspecific Candidate Gene Approach. MPMI.

[108] Moore L.D., Le T., Fan G. (2013). DNA Methylation and Its Basic Function. Neuropsychopharmacology.

[109] Xia Z., Zou M., Zhang S., Feng B., Wang W. (2014). AFSM Sequencing Approach: A Simple and Rapid Method for Genome-Wide SNP and Methylation Site Discovery and Genetic Mapping. Sci. Rep..

[110] Drozda A., Kurpisz B., Guan Y., Arasimowicz-Jelonek M., Plich J., Jagodzik P., Kuźnicki D., Floryszak-Wieczorek J. (2022). Insights into the Expression of DNA (de)Methylation Genes Responsive to Nitric Oxide Signaling in Potato Resistance to Late Blight Disease. Front. Plant Sci..

[111] Kuźnicki D., Meller B., Arasimowicz-Jelonek M., Braszewska-Zalewska A., Drozda A., Floryszak-Wieczorek J. (2019). BABA-Induced DNA Methylome Adjustment to Intergenerational Defense Priming in Potato to *Phytophthora infestans*. Front. Plant Sci..

[112] Saeed F., Dangol S.D., Hashmi M.H., Hossain M.J., Bakhsh A., Çalişkan M.E., Bakhsh A., Jabran K. (2023). Chapter 16—Role of Genetic Engineering in Improving Potato Production. Potato Production Worldwide.

[113] Haverkort A.J., Struik P.C., Visser R.G.F., Jacobsen E. (2009). Applied Biotechnology to Combat Late Blight in Potato Caused by *Phytophthora infestans*. Potato Res..

[114] del Mar Martínez-Prada M., Curtin S.J., Gutiérrez-González J.J. (2021). Potato Improvement through Genetic Engineering. GM Crops Food.

[115] Holme I.B., Wendt T., Holm P.B. (2013). Intragenesis and Cisgenesis as Alternatives to Transgenic Crop Development. Plant Biotechnol. J..

[116] Angmo D., Sharma S.P., Kalia A. (2023). Breeding Strategies for Late Blight Resistance in Potato Crop: Recent Developments. Mol. Biol. Rep..

[117] Halterman D., Guenthner J., Collinge S., Butler N., Douches D. (2015). Biotech Potatoes in the 21st Century: 20 Years Since the First Biotech Potato. Am. J. Potato Res..

[118] Jones J.D.G., Witek K., Verweij W., Jupe F., Cooke D., Dorling S., Tomlinson L., Smoker M., Perkins S., Foster S. (2014). Elevating Crop Disease Resistance with Cloned Genes. Philos. Trans. R. Soc. Lond. B Biol. Sci..

[119] Ghislain M., Byarugaba A.A., Magembe E., Njoroge A., Rivera C., Román M.L., Tovar J.C., Gamboa S., Forbes G.A., Kreuze J.F. (2019). Stacking Three Late Blight Resistance Genes from Wild Species Directly into African Highland Potato Varieties Confers Complete Field Resistance to Local Blight Races. Plant Biotechnol. J..

[120] Magembe E., Kariuki D., Webi E., Njoroge A., Ghislain M. (2019). Extreme Resistance to Late Blight Disease by Transferring 3 R Genes from Wild Relatives into African Farmer-Preferred Potato Varieties. Afr. J. Biotechnol..

[121] Executive Summary: Global Status of Commercialized Biotech/GM Crops: 2014—ISAAA Brief 49-2014|ISAAA.Org. https://www.isaaa.org/resources/publications/briefs/49/executivesummary/default.asp.

[122] Curtis K., Mccluskey J., Wahl T. (2002). Is China the Market for Genetically Modified Potatoes?. AgBioForum.

[123] Ambarwati A., Santoso T., Kusmana K. (2022). Experience in Developing Genetically Engineered Potato Resistance to Late Blight. https://www.researchgate.net/publication/363331329_Experience_in_developing_genetically_engineered_potato_resistance_to_Late_blight.

[124] Shin M.K., Jeon S.M., Koo Y.E. (2023). Detection Method for Genetically Modified Potato Using an Ultra-Fast PCR System. Food Sci. Biotechnol..

[125] Haverkort A.J., Boonekamp P.M., Hutten R., Jacobsen E., Lotz L.A.P., Kessel G.J.T., Vossen J.H., Visser R.G.F. (2016). Durable Late Blight Resistance in Potato Through Dynamic Varieties Obtained by Cisgenesis: Scientific and Societal Advances in the DuRPh Project. Potato Res..

[126] Helgeson J.P., Pohlman J.D., Austin S., Haberlach G.T., Wielgus S.M., Ronis D., Zambolim L., Tooley P., McGrath J.M., James R.V. (1998). Somatic Hybrids between *Solanum bulbocastanum* and Potato: A New Source of Resistance to Late Blight. Appl. Genet..

[127] Rakosy-Tican E., Thieme R., König J., Nachtigall M., Hammann T., Denes T.-E., Kruppa K., Molnár-Láng M. (2020). Introgression of Two Broad-Spectrum Late Blight Resistance Genes, Rpi-Blb1 and *Rpi-Blb3*, From *Solanum bulbocastanum* Dun Plus Race-Specific R Genes Into Potato Pre-Breeding Lines. Front. Plant Sci..

[128] Thieme R., Darsow U., Rakosy-Tican L., Kang Z., Gavrilenko T., Antonova O., Heimbach U., Thieme T. (2004). Use of Somatic Hybridisation to Transfer Resistance to Late Blight and Potato Virus Y (PVY) into Cultivated Potato. Plant Breed. Seed Sci..

[129] Shi Y.Z., Chen Q., Li H.Y., Beasley D., Lynch D.R. (2006). Somatic Hybridization between *Solanum tuberosum* and *S. cardiophyllum*. Can. J. Plant Sci..

[130] Sedlák P., Sedláková V., Vašek J., Zeka D., Čílová D., Melounová M., Orsák M., Domkářová J., Doležal P., Vejl P. (2022). Phenotypic, Molecular and Biochemical Evaluation of Somatic Hybrids between *Solanum tuberosum* and *S. bulbocastanum*. Sci. Rep..

[131] Sun K., Wolters A.-M.A., Vossen J.H., Rouwet M.E., Loonen A.E.H.M., Jacobsen E., Visser R.G.F., Bai Y. (2016). Silencing of Six Susceptibility Genes Results in Potato Late Blight Resistance. Transgenic Res..

[132] Kieu N.P., Lenman M., Wang E.S., Petersen B.L., Andreasson E. (2021). Mutations Introduced in Susceptibility Genes through CRISPR/Cas9 Genome Editing Confer Increased Late Blight Resistance in Potatoes. Sci. Rep..

[133] Sanju S., Siddappa S., Thakur A., Shukla P.K., Srivastava N., Pattanayak D., Sharma S., Singh B.P. (2015). Host-Mediated Gene Silencing of a Single Effector Gene from the Potato Pathogen *Phytophthora infestans* Imparts Partial Resistance to Late Blight Disease. Funct. Integr. Genom..

[134] Sun K., Wolters A.-M.A., Loonen A.E.H.M., Huibers R.P., van der Vlugt R., Goverse A., Jacobsen E., Visser R.G.F., Bai Y. (2016). Down-Regulation of Arabidopsis DND1 Orthologs in Potato and Tomato Leads to Broad-Spectrum Resistance to Late Blight and Powdery Mildew. Transgenic Res..

[135] Bradshaw J.E., Bradshaw J.E. (2021). Gene Editing and Genetic Transformation of Potatoes. Potato Breeding: Theory and Practice.

[136] Listanto E., Riyanti E.I., Ambarwati A.D. (2020). Transformation Using RNAi Technology for Developing Potato Lines Resistance to Late Blight (*Phytophthora infestans*). IOP Conf. Ser. Earth Environ. Sci..

[137] Hameed A., Tahir M.N., Asad S., Bilal R., Van Eck J., Jander G., Mansoor S. (2017). RNAi-Mediated Simultaneous Resistance against Three RNA Viruses in Potato. Mol. Biotechnol..

[138] Kim D.H., Rossi J.J. (2008). RNAi Mechanisms and Applications. BioTechniques.

[139] Padilla-Roji I., Ruiz-Jiménez L., Bakhat N., Vielba-Fernández A., Pérez-García A., Fernández-Ortuño D. (2023). RNAi Technology: A New Path for the Research and Management of Obligate Biotrophic Phytopathogenic Fungi. Int. J. Mol. Sci..

[140] Schaefer L.K., Parlange F., Buchmann G., Jung E., Wehrli A., Herren G., Müller M.C., Stehlin J., Schmid R., Wicker T. (2020). Cross-Kingdom RNAi of Pathogen Effectors Leads to Quantitative Adult Plant Resistance in Wheat. Front. Plant Sci..

[141] Wang X., Zhai T., Zhang X., Tang C., Zhuang R., Zhao H., Xu Q., Cheng Y., Wang J., Duplessis S. (2021). Two Stripe Rust Effectors Impair Wheat Resistance by Suppressing Import of Host Fe–S Protein into Chloroplasts. Plant Physiol..

[142] Jahan S.N., Åsman A.K.M., Corcoran P., Fogelqvist J., Vetukuri R.R., Dixelius C. (2015). Plant-Mediated Gene Silencing Restricts Growth of the Potato Late Blight Pathogen *Phytophthora infestans*. J. Exp. Bot..

[143] Latijnhouwers M., Govers F. (2003). A *Phytophthora infestans* G-Protein β Subunit Is Involved in Sporangium Formation. Eukaryot Cell.

[144] Van Den Hoogen J., Verbeek-de Kruif N., Govers F. (2018). The G-Protein γ Subunit of *Phytophthora infestans* Is Involved in Sporangial Development. Fungal Genet. Biol..

[145] Situ J., Xi P., Lin L., Huang W., Song Y., Jiang Z., Kong G. (2022). Signal and Regulatory Mechanisms Involved in Spore Development of Phytophthora and Peronophythora. Front. Microbiol..

[146] Kalyandurg P.B., Sundararajan P., Dubey M., Ghadamgahi F., Zahid M.A., Whisson S.C., Vetukuri R.R. (2021). Spray-Induced Gene Silencing as a Potential Tool to Control Potato Late Blight Disease. Phytopathology®.

[147] Whisson S.C., Avrova A.O., Van West P., Jones J.T. (2005). A Method for Double-Stranded RNA-Mediated Transient Gene Silencing in *Phytophthora infestans*. Mol. Plant Pathol..

[148] Sundaresha S., Sharma S., Bairwa A., Tomar M., Kumar R., Bhardwaj V., Jeevalatha A., Bakade R., Salaria N., Thakur K. Spraying of dsRNA Molecules Derived from Phytophthora infestans, along with Nanoclay Carriers as a Proof of Concept for Developing Novel Protection Strategy for Potato Late Blight.|Pest Management Science|EBSCOhost. https://openurl.ebsco.com/contentitem/doi:10.1002%2Fps.6949?sid=ebsco:plink:crawler&id=ebsco:doi:10.1002%2Fps.6949.

[149] Ivanov A.A., Golubeva T.S. (2023). Exogenous dsRNA-Induced Silencing of the *Phytophthora infestans* Elicitin Genes *Inf1* and *Inf4* Suppresses Its Pathogenicity on Potato Plants. J. Fungi.

[150] Porwal V., Sharma A., Kant A. (2020). Efficacy of Ds RNA in Late Blight (Phytophthora infestans) of Tomato. http://www.ir.juit.ac.in:8080/jspui/bitstream/123456789/6787/1/Efficacy%20of%20ds%20RNA%20in%20Late%20Blight-Phytophthora%20Infestans-%20of%20Tomato.pdf.

[151] Zhu H., Li C., Gao C. (2020). Applications of CRISPR–Cas in Agriculture and Plant Biotechnology. Nat. Rev. Mol. Cell Biol..

[152] Arora L., Narula A. (2017). Gene Editing and Crop Improvement Using CRISPR-Cas9 System. Front. Plant Sci..

[153] Doudna J.A., Charpentier E. (2014). Genome Editing. The New Frontier of Genome Engineering with CRISPR-Cas9. Science.

[154] He Y., Wang R., Dai X., Zhao Y., Weeks D.P., Yang B. (2017). Chapter Nine-On Improving CRISPR for Editing Plant Genes: Ribozyme-Mediated Guide RNA Production and Fluorescence-Based Technology for Isolating Transgene-Free Mutants Generated by CRISPR. Progress in Molecular Biology and Translational Science.

[155] Feng Z., Zhang B., Ding W., Liu X., Yang D.-L., Wei P., Cao F., Zhu S., Zhang F., Mao Y. (2013). Efficient Genome Editing in Plants Using a CRISPR/Cas System. Cell Res..

[156] Devi R., Chauhan S., Dhillon T.S. (2022). Genome Editing for Vegetable Crop Improvement: Challenges and Future Prospects. Front. Genet..

[157] Kusano H., Ohnuma M., Mutsuro-Aoki H., Asahi T., Ichinosawa D., Onodera H., Asano K., Noda T., Horie T., Fukumoto K. (2018). Establishment of a Modified CRISPR/Cas9 System with Increased Mutagenesis Frequency Using the Translational Enhancer dMac3 and Multiple Guide RNAs in Potato. Sci. Rep..

[158] Hegde N., Joshi S., Soni N., Kushalappa A.C. (2021). The Caffeoyl-CoA O-Methyltransferase Gene SNP Replacement in Russet Burbank Potato Variety Enhances Late Blight Resistance through Cell Wall Reinforcement. Plant Cell Rep..

[159] Ah-Fong A.M.V., Boyd A.M., Matson M.E.H., Judelson H.S. (2021). A Cas12a-Based Gene Editing System for *Phytophthora infestans* Reveals Monoallelic Expression of an Elicitor. Mol. Plant Pathol..

[160] Moon K.-B., Park S.-J., Park J.-S., Lee H.-J., Shin S.Y., Lee S.M., Choi G.J., Kim S.-G., Cho H.S., Jeon J.-H. (2022). Editing of StSR4 by Cas9-RNPs Confers Resistance to *Phytophthora infestans* in Potato. Front. Plant Sci..

[161] Bi W., Liu J., Li Y., He Z., Chen Y., Zhao T., Liang X., Wang X., Meng X., Dou D. (2024). CRISPR/Cas9-Guided Editing of a Novel Susceptibility Gene in Potato Improves *Phytophthora* Resistance without Growth Penalty. Plant Biotechnol. J..

[162] Yang L., McLellan H., Naqvi S., He Q., Boevink P.C., Armstrong M., Giuliani L.M., Zhang W., Tian Z., Zhan J. (2016). Potato NPH3/RPT2-like Protein StNRL1, Targeted by a *Phytophthora infestans* RXLR Effector, Is a Susceptibility Factor. Plant Physiol..

[163] Nourozi M., Nazarain-Firouzabadi F., Ismaili A., Ahmadvand R., Poormazaheri H. (2024). CRISPR/Cas StNRL1 Gene Knockout Increases Resistance to Late Blight and Susceptibility to Early Blight in Potato. Front. Plant Sci..

[164] Hou X., Guo X., Zhang Y., Zhang Q. (2023). CRISPR/Cas Genome Editing System and Its Application in Potato. Front. Genet..

[165] Dormatey R., Sun C., Ali K., Coulter J.A., Bi Z., Bai J. (2020). Gene Pyramiding for Sustainable Crop Improvement against Biotic and Abiotic Stresses. Agronomy.

[166] Malav A.K., Indu, Chandrawat K.S. (2016). Gene Pyramiding: An Overview. Int. J. Curr. Res. Biosci. Plant Biol..

[167] Mundt C.C. (2018). Pyramiding for Resistance Durability: Theory and Practice. Phytopathology.

[168] Taoutaou A., Berindean I.V., Chemmam M.K., Beninal L., Rida S., Khelifi L., Bouznad Z., Racz I., Ona A., Muntean L. (2023). Defeated Stacked Resistance Genes Induce a Delay in Disease Manifestation in the Pathosystem *Solanum tuberosum*—*Phytophthora infestans*. Agronomy.

[169] Torres Ascurra Y.C., Wouters D., Visser R.G.F., Nürnberger T., Vleeshouwers V.G.A.A. (2023). Stacking of PRRs in Potato to Achieve Enhanced Resistance against *Phytophthora infestans*. bioRxiv.

[170] Blatnik E., Horvat M., Berne S., Humar M., Dolničar P., Meglič V. (2022). Late Blight Resistance Conferred by *Rpi-Smira2/R8* in Potato Genotypes In Vitro Depends on the Genetic Background. Plants.

[171] Plich J., Tatarowska B., Lebecka R., Śliwka J., Zimnoch-Guzowska E., Flis B. (2015). *R2*-like Gene Contributes to Resistance to *Phytophthora infestans* in Polish Potato Cultivar Bzura. Am. J. Potato Res..

[172] Tan M.Y.A., Hutten R.C.B., Visser R.G.F., Eck H.J. (2010). van The Effect of Pyramiding *Phytophthora infestans* Resistance Genes *R Pi-Mcd1* and *R Pi-Ber* in Potato. Theor. Appl. Genet..

[173] Oh S., Kim S., Park H.-J., Kim M.-S., Seo M.-K., Wu C.-H., Lee H.-A., Kim H.-S., Kamoun S., Choi D. (2023). Nucleotide-Binding Leucine-Rich Repeat Network Underlies Nonhost Resistance of Pepper against the Irish Potato Famine Pathogen *Phytophthora infestans*. Plant Biotechnol. J..

[174] Brown-Donovan K.M. (2020). Pyramiding Approaches for Potato Disease Resistance Breeding.

[175] Taoutaou A., Socaciu C., Pamfil D., Balazs E., Botez C. (2011). Response of an *R* Gene Pyramided Potato Genotype to Infection with *Phytophthora infestans*. Bull. USAMV Agric..

[176] Taoutaou A., Socaciu C., Pamfil D., Balazs E., Botez C. (2013). Role of Some Phenolic Compounds in a Resistant Gene Pyramided Potato Genotype to Late Blight. Bulg. J. Agric. Sci..

[177] Brown J.K.M., Rant J.C. (2013). Fitness Costs and Trade-Offs of Disease Resistance and Their Consequences for Breeding Arable Crops. Plant Pathol..

[178] Bradshaw J.E., Vreugdenhil D., Bradshaw J., Gebhardt C., Govers F., MacKerron D.K.L., Taylor M.A., Ross H.A. (2007). Potato-Breeding Strategy. Potato Biology and Biotechnology: Advances and Perspectives.

[179] Haas B.J., Kamoun S., Zody M.C., Jiang R.H.Y., Handsaker R.E., Cano L.M., Grabherr M., Kodira C.D., Raffaele S., Torto-Alalibo T. (2009). Genome Sequence and Analysis of the Irish Potato Famine Pathogen *Phytophthora infestans*. Nature.

[180] Babarinde S., Burlakoti R.R., Peters R.D., Al-Mughrabi K., Novinscak A., Sapkota S., Prithiviraj B. (2024). Genetic Structure and Population Diversity of *Phytophthora infestans* Strains in Pacific Western Canada. Appl. Microbiol. Biotechnol..

[181] Shen L.-L., Waheed A., Wang Y.-P., Nkurikiyimfura O., Wang Z., Yang L., Zhan J. (2021). Multiple Mechanisms Drive the Evolutionary Adaptation of *Phytophthora infestans* Effector Avr1 to Host Resistance. J. Fungi—Open Access Mycol. J..

[182] Yang L.-N., Ouyang H., Nkurikiyimfura O., Fang H., Waheed A., Li W., Wang Y.-P., Zhan J. (2022). Genetic Variation along an Altitudinal Gradient in the *Phytophthora infestans* Effector Gene Pi02860. Front. Microbiol..

[183] Yang K., Yan Q., Wang Y., Zhu W., Wang X., Li X., Peng H., Zhou Y., Jing M., Dou D. (2023). Engineering Crop Phytophthora Resistance by Targeting Pathogen-Derived PI3P for Enhanced Catabolism. Plant Commun..

[184] Li Q., Wang B., Yu J., Dou D. (2021). Pathogen-Informed Breeding for Crop Disease Resistance. J. Integr. Plant Biol..

[185] Cook D.E., Mesarich C.H., Thomma B.P.H.J. (2015). Understanding Plant Immunity as a Surveillance System to Detect Invasion. Annu. Rev. Phytopathol..

[186] Mann C.W.G., Sawyer A., Gardiner D.M., Mitter N., Carroll B.J., Eamens A.L. (2023). RNA-Based Control of Fungal Pathogens in Plants. IJMS.

[187] Koch A., Wassenegger M. (2021). Host-Induced Gene Silencing-Mechanisms and Applications. New Phytol..

[188] Cai Q., Qiao L., Wang M., He B., Lin F.-M., Palmquist J., Huang S.-D., Jin H. (2018). Plants Send Small RNAs in Extracellular Vesicles to Fungal Pathogen to Silence Virulence Genes. Science.

[189] Ellendorff U., Fradin E.F., De Jonge R., Thomma B.P.H.J. (2009). RNA Silencing Is Required for Arabidopsis Defence against Verticillium Wilt Disease. J. Exp. Bot..

[190] Guo N., Zhao J., Yan Q., Huang J., Ma H., Rajput N.A., Jiang H., Xing H., Dou D. (2018). Resistance to Phytophthora Pathogens Is Dependent on Gene Silencing Pathways in Plants. J. Phytopathol..

